# A bacterial toxin as a novel anti-cancer drug modulating the tumor-microenvironment

**DOI:** 10.1038/s41419-025-08219-2

**Published:** 2025-12-01

**Authors:** Lingyu Li, Pauline Evain, Michael Timothy Phillips, Maria Lopez Chiloeches, Anna Bergonzini, Teresa Frisan, Sun Nyunt Wai, Saskia Friederike Erttmann

**Affiliations:** 1 Department of Molecular Biology, Umeå Centre for Microbial Research (UCMR), Umeå University, SE-90187 Umeå, Sweden; 2 The Laboratory for Molecular Infection Medicine Sweden (MIMS), Umeå University, SE-90187 Umeå, Sweden; 3 Laboratory of Infection Oncology, Institute of Clinical Molecular Biology, University of Kiel and University Hospital Schleswig Holstein (UKSH), 24105 Kiel, Germany

**Keywords:** Cancer microenvironment, Tumour immunology, Colorectal cancer

## Abstract

Colorectal cancer (CRC), the third-most prevalent and second deadliest cancer, requires new therapeutic strategies due to the significant side effects of current treatments. We investigated the anticancer properties of MakA, a cytotoxin from *Vibrio cholerae*, administered systemically in a mouse model, with a focus on its impact on the tumor microenvironment (TME) and immune cell infiltration. Our findings demonstrate that MakA administration is non-toxic and does not cause systemic tissue damage. It increases immune cell abundance in the TME, suppresses tumor growth, promotes cancer cell apoptosis, and enhances leukocyte recruitment and activation. Elevated neutrophil and macrophage densities were associated with increased production of pro-inflammatory mediators with anti-neoplastic properties. These findings highlight MakA’s potential as a targeted, less harmful CRC therapy by modulating the TME immune response.

## Introduction

Colorectal cancer is the third most prevalent cancer globally, accounting for ~10% of all cancer diagnoses, and is the second leading cause of cancer-related deaths worldwide [1]. Current treatment modalities, including surgery, chemotherapy, and radiotherapy, are often associated with significant side effects [2]. Recent advances in oncology have highlighted the therapeutic potential of bacteria, which can produce a variety of cytotoxic compounds, toxins, and enzymes capable of modifying prodrugs [3]. Bacterial toxins are recognized for their anticancer properties, as they can modulate key cellular processes such as apoptosis, differentiation, and proliferation [4–9]. These properties position bacterial toxins as promising candidates for the development of novel, effective, targeted, and safer anticancer therapeutics.

Our previous research identified motility-associated killing factor A (MakA), as a cytotoxin essential for the cytotoxic effects of *V. cholerae* in both *C. elegans* and *Danio rerio* (zebrafish) [10]. Studies of the *mak* gene cluster in *V. cholerae* have shown that MakA can assemble with MakB and MakE into a tripartite α-pore-forming toxin (α-PFT) complex, which plays a pivotal role in bacterial virulence and in interaction with diverse hosts and environmental contexts [11]. In addition, MakA induces the formation of tube-like structures in lipid membranes under acidic conditions, with cryo-electron microscopy revealing a unique protein-lipid superstructure within MakA filaments [12]. Recent studies have shown MakA’s capacity to bind to cell membranes, inducing cancer cell death through mechanisms such as autophagy modulation, apoptosis induction, and suppression of cell proliferation [13–16]. Furthermore, direct intratumoral administration of MakA markedly inhibited tumor growth in a mouse model of colorectal cancer, underscoring its therapeutic potential [15]. However, the specific effects of MakA on cancer progression, the tumor microenvironment (TME), and its safety when administered systemically remains to be fully elucidated.

The interaction between cancer cells and tumor-infiltrating leukocytes, particularly innate immune cells such as neutrophils and monocytes, plays a critical role in tumor progression [17–20]. Macrophages, derived from monocytes, are a dominant immune cell population within the TME and can modulate the activity of cytotoxic T lymphocytes and natural killer (NK) cells, thereby influencing patient outcomes [21–25]. In this study, we evaluated the anticancer properties of MakA in an allograft mouse model via systemic administration, focusing on immune cell infiltration within the TME. MakA was well-tolerated, promoted the recruitment of monocytes and neutrophils, and suppressed tumor growth by enhancing macrophage accumulation and stimulating these cells to secrete pro-inflammatory mediators, thereby inducing M1-like macrophage anti-tumor effects. These findings support MakA’s potential as a novel anticancer therapeutic targeting immune responses within the TME, particularly for colorectal cancer treatment.

## Results

### Systemic delivery of MakA does not cause toxicity

In our recent studies, intra-tumoral administration of MakA significantly inhibited tumor growth in a murine allograft colon cancer model, highlighting its potential as a novel therapeutic for colon cancer [15]. However, the safety of MakA following systemic administration (intravenous or intraperitoneal) had not been evaluated. To address this, we intravenously injected C57BL/6 mice with increasing doses of MakA and monitored clinical symptoms (Supplementary Fig. S1A). Our observations revealed that MakA had no effect on clinical severity, body temperature, or body weight at any of the dosages tested (Supplementary Fig. S1B–D). To further evaluate safety, NOD/SCID mice —chosen for their suitability in hosting slow-growing tumors due to their B- and T- cell deficiencies [26]—were intravenously injected with MakA four times, every third day (Supplementary Fig. S1E). These mice also exhibited no significant changes in clinical symptoms following MakA treatment (Supplementary Fig. S1F–H). Together, these findings demonstrate that systemic delivery of MakA is well-tolerated and does not induce detectable toxicity.

### MakA mediates monocyte and neutrophil recruitment into the peritoneal cavity

**Fig. 1:**
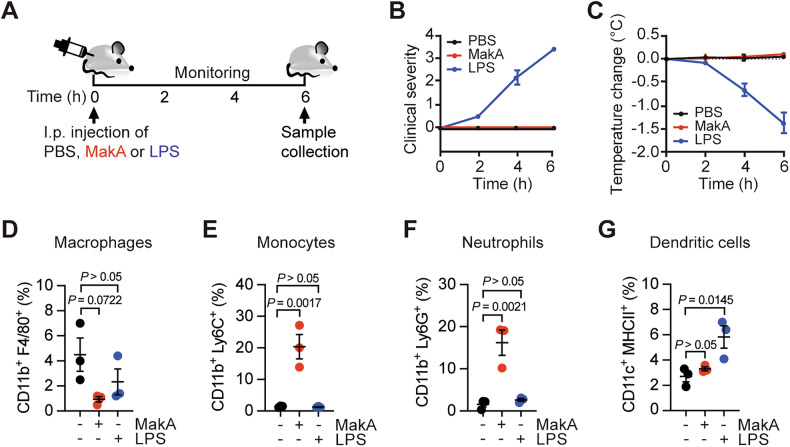
Effect of MakA on immune cell populations in the peritoneal cavity. Related to Supplementary Figs. S1–S5. **A** Experimental layout of the intraperitoneal injection of 2.5 mg kg^-1^ MakA, 10 mg kg^-1^ lipopolysaccharide (LPS), or PBS in C57BL/6 mice used in (**B**–**G**). **B** Clinical severity and **C** body temperature changes, *n* = 3. **D** Quantification (%) of flow cytometric analysis of macrophages (CD11b^+^ F4/80^+^ cells) in total peritoneal exudate cells (PECs). **E** Quantification (%) of flow cytometric analysis of monocytes (CD11b^+^ Ly6C^+^ cells) in total PECs. **F** Quantification (%) of flow cytometric analysis of neutrophils (CD11b^+^ Ly6G^+^ cells) in total PECs. **G** Quantification (%) of flow cytometric analysis of dendritic cells (CD11c^+^ MHCII^+^ cells) in total PECs. Data in (**B**–**G**) are presented as means ± standard error of the mean (SEM) from one experiment with *n* = 3 mice per group.

Understanding the properties and roles of peritoneal macrophages and their monocyte progeny is important for developing therapeutic strategies targeting inflammatory conditions and abdominal malignancies [27]. To assess the effects of MakA on immune cell recruitment, we administered MakA or lipopolysaccharide (LPS) intraperitoneally to C57BL/6 mice (Fig. 1A) and analyzed immune cell recruitment. Our findings showed that MakA administration did not provoke any adverse clinical symptoms, like the effects observed with PBS injections, confirming its non-toxicity in mice. Conversely, mice receiving LPS injections displayed a greater degree of clinical severity and hypothermia compared to those of the PBS and MakA treated group (Fig. 1B, C). Flow cytometric analyses revealed that MakA treatment decreased the percentage of macrophages with respect to the total number of cells within the peritoneal cavity (Fig. 1D, Supplementary Figs. S2A, S3A, B), suggesting that MakA alters the cellular composition of the peritoneal cavity by reducing the proportion of macrophages and favoring the infiltration of other immune cell types. Specifically, MakA enhanced the recruitment of monocytes (Fig. 1E, Supplementary Figs. S2B, S3C) and neutrophils (Fig. 1F, Supplementary Figs. S2C, S3D), whereas LPS did not significantly affect the recruitment of those immune cells into the peritoneal cavity (Fig. 1D–F, Supplementary Fig. S2A–C). In contrast, the proportion of dendritic cells (DCs) among the total peritoneal exudate cells remained unchanged after MakA treatment. However, LPS injection led to a higher percentage of DCs compared to the PBS control group (Fig. 1G, Supplementary Figs. S2D, S3E). Additionally, MakA did not alter the abundance of immune cells in spleens, whereas LPS caused a slight decrease in splenic monocyte, neutrophil, CD4^+^, and CD8^+^T cell percentages (Supplementary Figs. S4A–H, S5A–C). Collectively, these findings indicate that MakA selectively recruits monocytes and neutrophils into the peritoneal cavity without triggering an inflammatory response in the spleen.

### MakA exhibits anti-tumor properties in the absence of tissue damage in mice

**Fig. 2:**
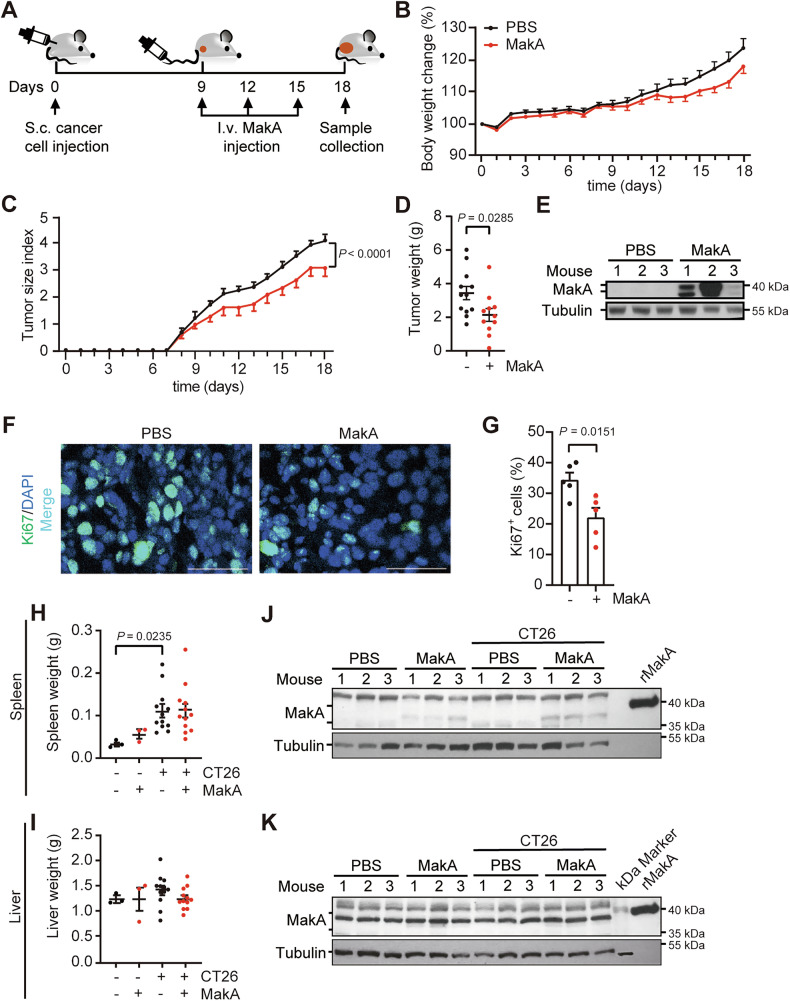
MakA exhibits anti-tumor efficacy by inhibiting cancer cell proliferation. Related to Supplementary Fig. S6. **A** Experimental layout of the allograft model using NOD/SCID mice subcutaneously injected with CT26 colon cancer cells and then sequentially treated with MakA (or PBS) every third day, used in (**B**–**K**). **B** Body weight change in mice, expressed in percent (*n* = 12 mice per group). **C** Quantification of tumor growth depicted as tumor size index. Data in (**B**) and (**C**) are means ± standard deviation (SD) (*n* = 12; from one experiment), *P* values determined by two-way ANOVA. **D** Weight of tumor tissue on day 18 (*n* = 12 mice per group). **E** Immunoblotting of MakA in tumor tissue lysates (*n* = 3). **F** Representative micrographs and **G** quantification of immunofluorescence staining for Ki67 (green) in tumor tissue of mice treated or not with MakA (*n* = 5). Scale bar: 50 μm. Weight of **H** spleens and **I** livers on day 18 (*n* = 3 non-tumor groups; *n* = 12 tumor groups). Immunoblots of MakA in **J** spleen and **K** liver lysates; three mice per group are depicted. rMakA denotes recombinant MakA control; kDa Marker indicates the protein ladder lane. Data are acquired from two independent experiments. Data in (**B**–**D**, **G**–**I**) are presented as mean ± SEM.

**Fig. 3:**
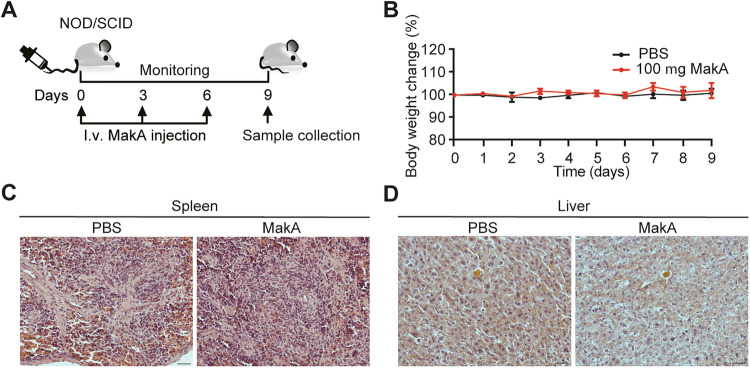
Systemic administration of MakA does not promote immune cell recruitment to spleen or liver. Related to Supplementary Fig. S6. **A** Experimental layout depicting NOD/SCID mice *i.v*. injected with 100 µg MakA (or PBS) on day 0, 3, and 6, and organ collection on day 9, used in (**B**–**D**). **B** Body weight change in percent. Data in (**B**) are presented as means ± SEM from one experiment with *n* = 3 mice per group. Representative light microscopy pictures of hematoxylin & eosin (H&E) stained 5-µm sections of **C** spleen and **D** liver at 20× magnification (*n* = 3 per group). Scale bar: 100 µm.

Our recent research has shown that MakA inhibits colon cancer cell growth by inducing apoptosis and exerting cytotoxic effects against various cancer cell lines, while displaying minimal toxicity toward non-transformed cells in vitro [15, 16]. Nonetheless, the mechanisms by which MakA affects tumor growth in vivo and its potential impact on the TME remain to be fully elucidated. To investigate this, NOD/SCID mice were subcutaneously inoculated with CT26 murine colon tumor cells and subsequently received intravenous (*i.v*.) injections of MakA or PBS control every third day for a total of three doses (Fig. 2A). Body weight changes in MakA-treated mice were comparable to those in PBS controls throughout the experimental period (Fig. 2B). Notably, MakA treatment significantly suppressed tumor growth, as indicated by reduced tumor size index and tumor weight (Fig. 2C, D). Efficient systemic delivery of MakA to the tumor was confirmed by the sustained presence of MakA protein in tumor tissues (Fig. 2E, Supplementary Fig. S11A). MakA administration also markedly decreased levels of the proliferation marker Ki67 in tumors, indicating reduced tumor cell proliferation (Fig. 2F, G). Importantly, MakA did not affect spleen or liver weights (Fig. 2H, I), likely due to the absence of MakA accumulation or efficient degradation in these organs (Fig. 2J, K, Supplementary Fig. S11B, C). Histological assessments showed no tissue damage or morphological changes in the spleen and liver (Fig. 3A–D). Although we hypothesized that MakA might localize to low pH environments, it was not detected in the acidic regions of the stomach and duodenum (Supplementary Figs. S6A, B, S12). Taken together, these findings demonstrate that systemic MakA administration effectively curtails tumor progression without causing collateral tissue damage, highlighting its potential as a safe and promising cancer therapeutic.

### MakA facilitates macrophage accumulation in the TME

**Fig. 4:**
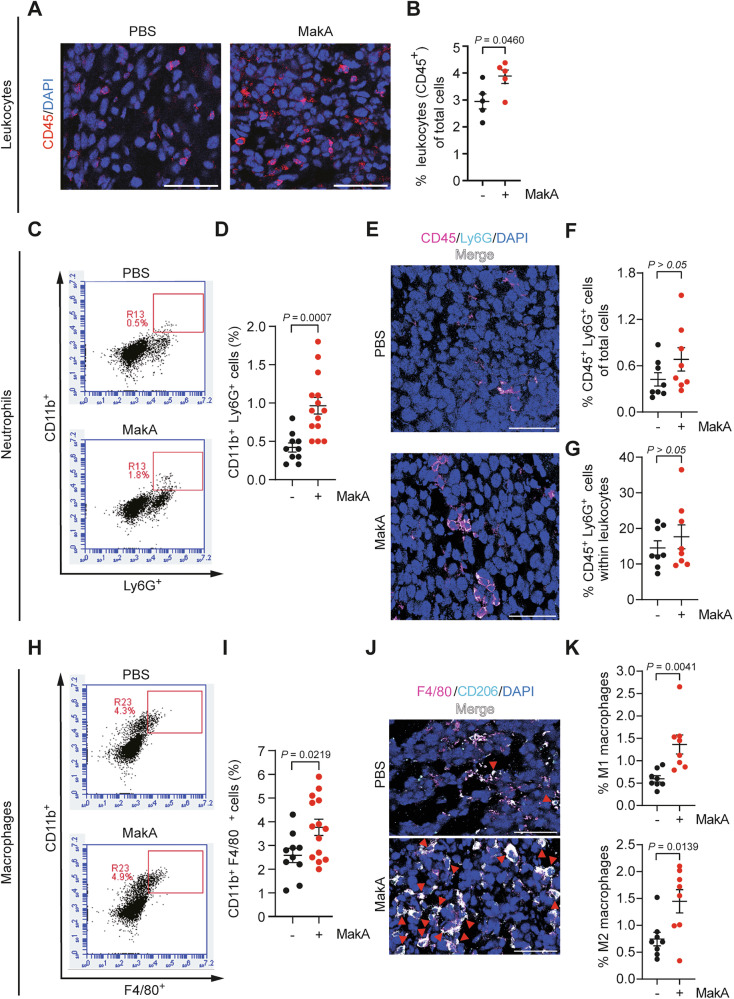
MakA mediates leukocyte accumulation, in particular macrophage enrichment in the TME. Related to Supplementary Fig. S7, S8. **A**–**K** Analysis of different cell populations in allograft tumors from mice *i.v*. injected with or without MakA. **A** Micrographs of IF staining for the leukocyte marker CD45 (red) with DAPI counterstaining. **B** IF quantification of leukocyte percentage in total tumor cells (*n* = 5 mice per group). **C** Representative flow cytometry plots of neutrophils (CD11b^+^ Ly6G^+^ cells) in tumors. **D** Flow cytometric quantification (%) of neutrophils in total tumor cells (*n* = 10 non-MakA groups; *n* = 14 MakA group). **E** Microscopic images of IF staining for CD45 (magenta) and neutrophil marker Ly6G (cyan) in tumors with DAPI counterstaining. Magenta (CD45^+^) and white-positive (CD45^+^ Ly6G^+^) cells indicate leukocytes and neutrophils, respectively. IF quantification (%) of neutrophils (CD45^+^ Ly6G^+^ cells) in **F** total tumor cells and **G** within leukocytes (*n* = 8 mice per group). **H** Representative flow cytometric plots of macrophages (CD11b^+^ F4/80^+^ cells) in tumors. **I** Flow cytometric quantification (%) of macrophages in total tumor cells (*n* = 10 non-MakA groups; *n* = 14 MakA group). **J** Micrographs of IF staining for total macrophage marker F4/80 (magenta) and M2 macrophage marker CD206 (cyan) in tumors with DAPI counterstaining. Magenta (F4/80^+^) and white-positive (F4/80^+^ CD206^+^) cells represent total macrophages and M2 macrophages, respectively. **K** IF quantification of M1- and M2-like macrophages (%) in total tumor cells (*n* = 8 mice per group). In (**A**, **E**, **J**), scale bar: 50 μm. In (**B**, **F**, **G**, **K**), each dot represents the mean count from 15 to 20 non-overlapping fields per section. Data are acquired from two independent experiments. Data in (**B**, **D**, **F**, **G**, **I**, **K**) are depicted as mean ± SEM.

Inflammatory cells and mediators are key components of the TME, with variations in cellular composition, cytokine networks, and molecular drivers across different tumor types. To investigate MakA’s role in immune cell recruitment to the TME, we performed immunofluorescence (IF) staining for CD45, a leukocyte marker, on tumor tissues. This analysis revealed increased leukocyte abundance in tumor from MakA-treated mice (Fig. 4A, B). The TME contains tumor cells alongside immune cells such as NK cells, DCs, neutrophils, and macrophages, which can either promote tumor progression or impede it via the secretion of cytokines and chemokines [19, 24]. To determine which immune cell type primarily drives MakA-induced leukocyte recruitment in the TME, we analyzed neutrophil and macrophage populations in allograft tumors by both immunofluorescence microscopy and flow cytometry. Flow cytometric analyses revealed a significant increase of CD11b^+^ Ly6G^+^ cells, indicative of neutrophils, following MakA treatment (Fig. 4C, D). IF staining for the neutrophil marker Ly6G^+^ confirmed these results, revealing a slight, but not significant, increase in the proportion of neutrophils (CD45^+^ Ly6G^+^ cells) among tumor cells in MakA-treated mice (Fig. 4E, F), as well as in the percentage of neutrophils within the leukocyte population (Fig. 4E, G). These findings collectively demonstrate that MakA does not substantially alter neutrophil abundance in the TME.

We next examined macrophage abundance within the TME, given their central role as mediators of inflammation and modulators of cancer progression. Tumor-associated macrophages (TAMs) are typically classified as anti-tumorigenic M1-like or pro-tumorigenic M2-like phenotypes, with the ability to adapt and transition in response to changes in the TEM or therapeutic interventions [18, 28]. Flow cytometric analyses and IF staining for F4/80^+^ cells revealed a marked increase in intratumoral macrophages in MakA-treated mice, suggesting enhanced recruitment of resident macrophages as well as monocytes followed by macrophage differentiation (Fig. 4H–K). Within the TME, MakA treatment was associated with an increased proportion of M2-like macrophages, as indicated by a higher frequency of CD206⁺ F4/80⁺ cells (Fig. 4J, K). In addition, the percentage of CD206^-^ F4/80^+^ cells, representing the M1-like subtype, was also significantly increased, indicative of a potentially pro-inflammatory and anti-tumorigenic microenvironment. Supporting these in vivo observations, MakA treatment significantly enhanced the polarization of M1-like macrophages in primary murine bone marrow-derived macrophages (BMDMs) in vitro (Supplementary Figs. S7A, B, S8). Taken together, these data demonstrate that MakA promotes the accumulation of both M1- and M2-like macrophages in the TME, without altering neutrophil abundance.

### MakA mediates the gene expression of immune modulators in the TME

**Fig. 5:**
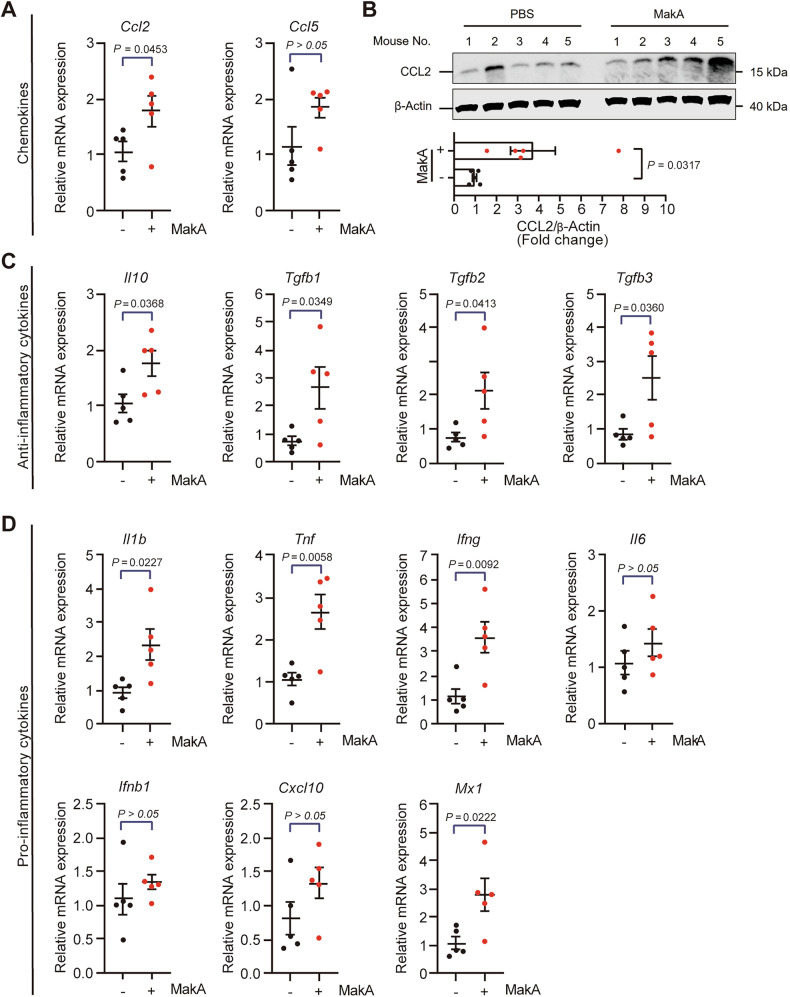
MakA strongly affects cytokine expression in the tumor microenvironment. Related to Supplementary Figs. S9, S10. **A**–**D** Gene expression profiles and protein levels of immune mediators in allograft tumors of mice *i.v*. injected with or without MakA. **A** mRNA levels of chemokines *Ccl2* and *Ccl5*, and **B** protein level of CCL2. **C** Anti-inflammatory cytokines *Il10, Tgfb1, Tgfb2*, and *Tgfb3*, and **D** pro-inflammatory mediators *Il1b, Tnf, Ifng, Il6, Ifnb1, Cxcl10*, and *Mx1*. Data are shown as mean ± SEM; *n* = 5 mice per group.

Chemokine gradients in the TME regulate immune cell migration and infiltration into solid tumors. Dysregulated chemokine signaling can promote tumor growth by excluding effector immune cells, driving immune cell suppression and exhaustion, and attracting additional immunosuppressive cells [23, 29, 30]. Using RT-qPCR and Western blot analyses, we found that MakA treatment significantly increased the mRNA expression of *Ccl2* and the protein level of CCL2 (also known as monocyte chemoattractant protein-1, MCP-1) in tumors of MakA treated mice (Fig. 5A, B, Supplementary Fig. S13A). Although *Ccl5* (also known as regulated on activation, normal T cell expressed and secreted, RANTES) expression was also elevated, this increase was not statistically significant (Fig. 5A). TAMs, by secreting a wide array of cytokines, play a pivotal role in shaping the TME by influencing cancer progression, metastasis, and the immune response. By interactions with both immune cells and tumor cells, TAMs contribute to the regulation of cancer cell plasticity [21, 25, 31]. To investigate cytokine profiles in relation to tumor growth inhibition, we assessed cytokine expression within the TME following MakA treatment. Gene expression analyses revealed elevated levels of the anti-inflammatory cytokines *Il10* and *Tgfb* (Fig. 5C). In line with these findings, increased protein levels of TGFβ-1 were detected (Supplementary Figs. S9A, B, S13B). When measuring mRNA levels of pro-inflammatory cytokines, including *Il1b*, *Tnf*, and *Ifng*, which contribute to the establishment of an M1-like microenvironment and exert positive feedback on unpolarized macrophages [18, 32], as well as type-I interferon stimulated genes (*Ifnb*, *Cxcl10*, *Mx1*), we detected a marked increase in these inflammatory mediators in the TME of MakA-treated mice (Fig. 5D). To determine the source of these cytokines, we analyzed their gene expression in macrophages and tumor cells following MakA treatment in vitro. In BMDMs, MakA significantly enhanced the expression of *Il1b*, *Tnf*, *Il6*, *Ifng*, *Ifnb1*, and *Cxcl10*, while decreasing *Tgfb3*. *Tgfb1*, *Tgfb2*, and *Il10* remained unaltered (Supplementary Fig. S10A). In MakA-treated CT26 colon cancer cells, most cytokines stayed undetectable except for marginally induced *Il6* and *Ifnb1* (Supplementary Fig. S10B), neither of which was significantly altered in vivo (Fig. 5D). Collectively, these results indicate that MakA does not trigger an inflammatory response in tumor cells but rather induces a selective cytokine and chemokine response in resident macrophages, leading to enhanced immune cell infiltration and an altered cytokine milieu within the TME.

### MakA induces macrophage-derived cytokines with pro-inflammatory and anti-tumorigenic properties

**Fig. 6:**
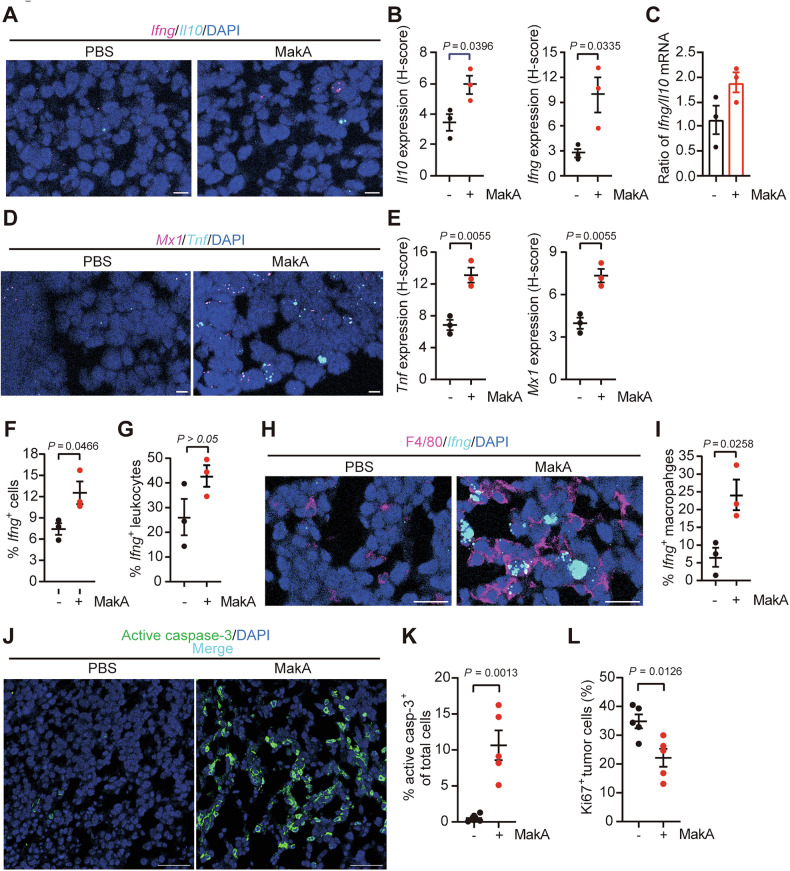
Macrophage-secreted cytokines contribute to MakA-induced tumor suppression. **A**–**L** IF and RNAscope multiplex fluorescent assay analyses on sections of allograft tumors from mice *i.v*. injected with or without MakA. **A** Representative micrographs of *Il10* and *Ifng* gene expression detected by in-situ transcriptomics (RNAscope). Cells are counterstained with DAPI, with each magenta and cyan spot depicting a single *Ifng* and *Il10* mRNA molecule, respectively. Scale bar: 10 μm. **B** Gene expression quantification of *Il10* and *Ifng* in tumors of mice detected by RNAscope analysis using H-Score. A single data point depicts the mean of 5 non-overlapping fields per section. **C** Ratio of *Ifng/Il10* mRNA in tumors of mice treated or not with MakA. **D** Representative micrographs of *Mx1* and *Tnf* gene expression detected by RNAscope. Cells are counterstained with DAPI, with each magenta and cyan spot depicting a single *Mx1* and *Tnf* mRNA molecule, respectively. Scale bar: 10 μm. **E** Gene expression quantification of *Tnf* and *Mx1* in tumors of mice detected by RNAscope analysis using H-Score. A single data point depicts the mean of 10 non-overlapping fields per section. **F**, **G** RNAscope analyses of *Ifng* combined (or not) with IF staining for leukocyte marker CD45 in tumors. Quantification in percent of *Ifng* positive cells (**F**) in tumors and (**G**) in *Ifng* positive leukocytes in total leukocytes in the TME. **H** Representative micrographs of RNAscope analyses of *Ifng* (cyan) combined with IF staining for macrophage marker F4/80 (magenta) in tumors. Scale bar: 20 μm. **I** Quantification in percent of *Ifng* positive macrophages in total macrophages in the TME. **J** Representative micrographs of IF staining for cleaved (active) caspase-3 (green) in tumors. Scale bar: 50 μm. **K** Quantification of cells positive for active caspase-3 in tumors (active casp-3^+^). **L** Quantification (%) of cancer cells positive for Ki67 in the TME (Ki67^+^ CD45^-^). Data in (**B**, **C**, **E**, **F**, **G**, **I**, **K**, **L**) are presented as mean ± SEM (*n* = 3 or 5 mice per group, respectively).

To investigate the contribution of macrophages to cytokine production in the TME, we performed RNAscope multiplex fluorescent assays on allograft tumors. MakA treatment significantly increased the expression of *Il10* and *Ifng* within tumors (Fig. 6A, B) and elevated the *Ifng/Il10* ratio (Fig. 6C), indicative of a more anti-tumorigenic cytokine milieu. In addition, mRNA levels of *Tnf* and *Mx1* (an interferon-stimulated gene linked to *Ifnb1* expression) were upregulated in MakA-treated tumors (Fig. 6D, E), suggesting a pro-inflammatory TME with enhanced anti-cancer potential. To determine the cellular sources of these cytokines, we assessed co-expression of *Ifng* with the leukocyte marker CD45 and the macrophage marker F4/80. This analysis revealed that MakA significantly increased the proportion of leukocytes, and in particularly macrophages, expressing *Ifng* (Fig. 6F–I), supporting a role for these immune cells in mediating anti-tumor activity. Consistent with these findings, immunofluorescence staining for cleaved caspase-3 demonstrated a higher proportion of apoptotic tumor cells in MakA-treated tumors compared with controls (Fig. 6J, K). Moreover, Ki67 staining showed reduced cancer cell proliferation in MakA-treated mice (Fig. 6L). Collectively, these findings indicate that MakA enhances cytokine production in the TME, particularly *Ifng* derived from macrophages, thereby contributing to tumor inhibition through increased apoptosis and decreased proliferation of cancer cells. Together, the data demonstrate that MakA reprograms the TME toward an inflammatory, anti-tumor state driven by macrophage activity.

## Discussion

Targeted cancer therapy using bacterial factors represents a promising strategy by exploiting bacterial products to selectively attack cancer cells and modulate the immune response, aiming for minimal side effects and improved efficacy [33]. In this study, we evaluated the anti-cancer properties of the MakA toxin through systemic administration in a mouse model. The NOD/SCID strain was selected because of its lack of functional T and B cells and reduced NK cell activity, allowing human-like tumor engraftment while preserving innate immune components such as macrophages and neutrophils. This feature provided an optimal platform to investigate MakA’s effects on the TME and innate immune modulation without interference from adaptive immunity.

Our findings demonstrate that MakA, even at higher dosages, exhibits a non-toxic profile, highlighting its potential as a safe therapeutic candidate for colon cancer and paving the way for future clinical studies. Systemically administered MakA accumulated within tumor tissues, where it reduced proliferation and promoted apoptosis of cancer cells, ultimately inhibiting tumor growth without compromising overall health.

Immune cell recruitment and functional modulation are key determinants of tumor progression or regression, particularly within the abdominal cavity [34]. We observed that MakA profoundly influenced immune cell dynamics by enhancing the recruitment of monocytes and neutrophils, which are pivotal in the innate immune response and inflammation. Importantly, these effects were restricted to the TME and not observed in the spleen, indicating a localized rather than systemic immune modulation. Such spatial specificity is crucial for therapeutic applications, as it ensures targeted anti-tumor activity while minimizing off-target immune activation. The TME represents a complex network comprising cancer cells, immune cells, extracellular matrix components, and signaling molecules that contribute to cancer progression and response to therapy [35]. Within this milieu, inflammatory cells and mediators can either promote tumor development or facilitate immune-mediated tumor clearance [36]. Consistent with this, MakA increased leukocyte recruitment, particularly neutrophils and macrophages, into allograft tumors, demonstarting active modulation of immune landscape. The functional balance between M1-like and M2-like macrophages critically influences cancer progression [24, 37]. While M1-like macrophages are generally associated with pro-inflammatory, anti-tumor functions such as cytokine production and promotion of cytotoxic immune responses, M2-like macrophages are typically linked to pro-tumor activities including angiogenesis, immune suppression, and tissue remodeling, although they may also exert anti-tumor effects under certain conditions [24, 37].

Interestingly, MakA increased both M1-like and M2-like macrophages within tumors, while in vitro it promoted a marked polarization of bone marrow–derived macrophages (BMDMs) toward the M1-like phenotype. This shift is likely driven by MakA-induced alterations in chemokine profiles within the TME. Chemokines are central to immune cell trafficking and functional programming. Dysregulated chemokine signaling can favor tumor growth by attracting immunosuppressive cell populations [29]. For example, CCL2 recruits monocytes to tumors, where they can differentiate into TAMs that support tumor growth and metastasis by releasing cytokines and growth factors [38]. However, recent studies indicate that CCL2 can also facilitate anti- tumor responses by enhancing monocyte and neutrophil functions [39], suggesting a context-dependent role of CCL2. Consistent with this, our data show that MakA significantly increased *Ccl2* mRNA expression in the TME, potentially enhancing monocyte/macrophage recruitment. Given the functional plasticity of macrophages, such recruitment could be redirected toward anti-tumor activities depending on local signaling cues [22]. Thus, MakA-driven CCL2 expression may represent a mechanism for reprogramming macrophage responses toward tumor inhibition. This context-dependent role of CCL2 in shaping macrophage function represents an interesting avenue for further investigation.

The observed upregulation of pro-inflammatory mediators (*Il1b*, *Tnf*, *Ifng*, and *Mx1*) alongside anti-inflammatory cytokines (*Il10* and *Tgfb*s) suggests that MakA orchestrates a dual immune program amplifying inflammatory, potentially tumoricidal responses while simultaneously inducing regulatory mechanisms to prevent excessive tissue damage [40]. The distinct cytokine profiles between macrophages and tumor cells after MakA treatment confirm that macrophages are the main source of these immune mediators in the TME. This is consistent with their established roles as key cytokine producers that modulate immune responses and interact with tumor cells [41, 42]. Notably, the reduced *Tgfb3* expression in macrophages further supports a shift toward a less immunosuppressive environment, given Tgf-β’s well-recognized role in promoting tumor growth and immune evasion [43]. Collectively, these findings demonstrate that MakA reshapes the chemokine and cytokine landscape, driving monocyte recruitment and macrophage polarization toward an anti-tumorigenic phenotype.

The intriguing observation that tumor growth was inhibited despite increased numbers of both M1- and M2-like macrophages may be explained by phenotypic reprogramming of TAMs. MakA may skew TAMs toward tumoricidal functions, possibly through IFNγ-mediated signaling, which can override the tumor-promoting effects typically associated with M2-like cells. The simultaneous elevation of pro-inflammatory and anti-inflammatory cytokines suggests a finely tune immune orchestration in which tumor-suppressive signals may temporarily override the tumor-promoting effects typically associated with M2-like macrophages. Additionally, MakA may exert direct anti-tumoral effects, as reported previously [15] or influence other components of TME in ways that remain to be fully defined. This complex interplay between MakA, the immunological landscape, and the TME underscores its potential for therapeutic exploitation and highlights the importance of a comprehensive understanding of immune reprogramming in cancer therapy.

## Materials and methods

### Mice

C57BL/6 (C57BL/6J; strain #:000664, RRID:IMSR_JAX:000664) mice were purchased from Jackson Laboratory, bred, and maintained under specific pathogen-free (SPF) conditions at the Umeå Centre for Comparative Biology (UCCB). NOD/SCID (NOD.CB17-Prkdcscid/J, strain code: 634) mice [26] were purchased from Charles River (France) and maintained under SPF conditions at UCCB. All animal experiments were performed at UCCB in accordance with the guidelines of the Umeå Regional Animal Ethic Committee. For experiments, male, co-housed 8–12-week-old C57BL/6 were used. For the allograft tumor model, male, co-housed NOD/SCID mice aged 6–8 weeks were used.

### Animal experiments

To determine the optimal injection dose of MakA, C57BL/6 mice were intravenously (*i.v*.) injected with 0, 10, 25, 50, or 100 μg of MakA in a total volume of 50 μl of Dulbecco’s Phosphate-Buffered Saline (DPBS; Gibco). After seven days, mice were euthanized. To initially assess potential toxicity of MakA in NOD/SCID mice, animals were *i.v*. injected with 100 μg MakA four times, every third day. Mice were then euthanized after a total of 14 days. To assess the effect of MakA on immune cell recruitment, C57BL/6 mice were intraperitoneally (*i.p*.) injected with either 2.5 mg MakA or 10 mg lipopolysaccharide (LPS) per kg body weight. Six hours post-infection, mice were euthanized, and peritoneal exudate cells (PECs) and spleens were collected for the immune cell recruitment assay by flow cytometric analysis. To investigate the influence of MakA on tumor progression, NOD/SCID mice were subcutaneously inoculated with 2 × 10^6^ CT26 colorectal cancer cells in 150 μL PBS. After nine days, when tumor growth became visible, mice were *i.v*. injected with 100 μg MakA in 50 μL DPBS or 50 μL DPBS as a control every third day for a total of three injections. Tumor growth (tumor size index) was monitored daily and assessed on an arbitrary scale from 1 to 5, where 1 indicated mice with small but visible tumors, and 5 represented mice with large tumors that required termination of mice. On day 18 of the experiment, mice were terminated, and tumors, livers, and spleens were weighed and collected for further analyses. In addition, stomach and duodenum tissues were collected for MakA abundance detection. Throughout all experiments, mice were monitored at least daily for clinical symptoms such as percentage of weight loss, changes in body temperature, and clinical severity. Clinical severity was scored on an arbitrary scale from 1 to 4, where 1 indicated mice with mild but visible symptoms such as reduced activity, and 4 represented those with the highest morbidity. High morbidity was characterized by combinations of hunched back posture, lethargy, loose fecal pellet, ruffled fur, prolonged hypothermia, ≥20% weight loss, presence of blood in stool, eyes, or anus, difficulties in breathing, impaired movement, behavioral interference due to tumors, or abdominal fluid accumulation causing stomach distension exceeding 10% of body weight.

### Cell culture and macrophage differentiation

CT26 colorectal cancer cells (CT26.WT; ATCC, CRL-2638; RRID:CVCL-7256) were cultured in RPMI 1640 Medium (Gibco, 11875093) supplemented with 10% heat-inactivated (HI)-fetal bovine serum (FBS) (Gibco, 26010066) and 1% penicillin-streptomycin (P/S; Gibco, 15140122) at 37 °C and 5% CO_2_. Mouse bone marrow-derived macrophages (BMDMs) were generated from C57BL/6 mice. Briefly, femurs and tibiae were isolated, and muscles and tendons were removed. Bone marrow cells were flushed from the bones with DPBS using a 27-Gauge 0.4 mm syringe needle. Red blood cells were lysed using RBC lysis buffer (155 mM NH_4_Cl, 12 mM NaHCO_3_, 0.1 mM EDTA). Subsequently, the remaining cells were washed with Iscove’s Modified Dulbecco’s Medium (IMDM, Gibco, 31980022) supplemented with 10% HI-FBS and 1% P/S, then seeded in non-cell culture treated petri dishes for differentiation in IMDM with 10% HI-FBS, 1% P/S and 20% M-CSF-containing conditioned medium from L929 cells for five days at 37 °C and 5% CO_2_.

To generate M0 macrophages, bone marrow cells were cultured under similar conditions in IMDM containing 10% HI-FBS, 50 µM β-mercaptoethanol and 100 ng/ml recombinant murine M-CSF (rmM-CSF, PeproTech, AF-315-02) in non-cell culture treated petri dishes for five days at 37 °C and 5% CO_2_.

### Cell stimulation

On day 5 of differentiation, BMDMs were seeded at a density of 1.5 × 10^6^ cells per well of a 6-well plate in IMDM supplemented with 10% HI-FBS. CT26 cells were seeded at a density of 0.6 × 10^6^ cells per well of a 6-well plate in RPMI supplemented with 10% HI-FBS. Twelve hours post-seeding, cells were washed twice with DPBS and then stimulated with either 250 or 500 nM MakA in serum-free medium for 12 h. Afterwards, cells were lysed in RNA lysis buffer (RLT; QIAGEN) containing 1% β-mercaptoethanol (Carl Roth, 60-24-2) for RNA isolation. To assess the effect of MakA on macrophage polarization, M0 macrophages were treated with or without 250 nM MakA for 24 h, and then cells were harvested for flow cytometric analysis.

### RNA isolation and quantitative reverse transcription polymerase chain reaction (RT-qPCR)

Tissue homogenates were prepared using a Bio-Gen PRO200 homogenizer (PRO Scientific) in RLT buffer containing 1% β-mercaptoethanol. Total RNA from tissues and cells was extracted using the RNeasy Mini Kit (QIAGEN, 74106) and the RNase-Free DNase Set (QIAGEN, 79256). cDNA synthesis was carried out with RevertAid H Minus Reverse Transcriptase (Thermo Fisher, EP0451) according to the manufacturer’s standard protocol with Random Hexamer Primer (Thermo Fisher, SO131) and Oligo(dT) 18 Primer (Thermo Fisher, SO142). Real-time qPCR was performed using QuantStudio 5 and analyzed by QuantStudio Design & Analysis software v1.4.3 (Applied Biosystems; Thermo Fisher Scientific). The qPCR conditions were 50°C for 2 min, 95°C for 10 min, and 45 cycles of 95°C for 15 s and 60°C for 1 min. The following TaqMan Gene Expression Assays (FAM/VIC), in combination with the TaqMan Gene Expression Master Mix were applied: *Il1b* (Mm004434228), *Il6* (Mm00446190), *Tnf* (Mm00443258), *Tgfb1* (Mm01178820), *Tgfb2* (Mm00436955), *Tgfb3* (Mm00436960), *Ifnb1* (Mm00439552), *Cxcl10* (Mm00445235), *Mx1* (Mm00487796), *Ifng* (Mm01168134), *Il10* (Mm00439615), *Ccl2* (Mm00441242), *Ccl5* (Mm01302427) and Rn18S; Rn4+ (Mm03928990). Results were normalized against 18S (VIC) and expressed as fold change relative to untreated (PBS) controls using the comparative CT method (2^-△△CT^).

### Western blot analysis

To detect selected proteins in various organs, including tumor, liver, spleen, stomach, and duodenum, tissues were homogenized in RIPA buffer (20 mM Tris HCl, pH 7.4; 150 mM NaCl; 1 mM EDTA; 1% NP-40; 0.2% SDS; 0.5% sodium deoxycholate) supplemented with 1× phosphatase inhibitor cocktail A and B (Biotool, B15001) and 1× protease inhibitor cocktail (Roche, 1836145001). Lysates were incubated on ice for 30 min and centrifuged at 10,000 × *g* for 15 min at 4°C. Supernatants were collected, and protein concentrations were determined using the BCA Protein Assay Kit (Thermo Scientific Pierce™ BCA protein assay, Waltham, MA). Samples were diluted with RIPA buffer as required, mixed with 2× Laemmli buffer containing 3% mercaptoethanol to a final concentration of 1.25 μg μL^−1^ and heated at 95°C for 5 min. Equal amount of protein lysates (25 μg) were subjected to electrophoresis on 10% or 15% SDS-PAGE gels and transferred onto Amersham Protran 0.45 μm NC nitrocellulose Western blotting membranes (Cytiva, 10600002) using a wet blot transfer system operating at 100 V on ice for 2 h. After protein transfer, membranes were blocked in 1× Roti Block buffer (Roth, A151.2) for 1 h at room temperature, then incubated overnight at 4°C under agitation with primary antibodies: MakA antiserum [10] (Agrisera AB), CCL2 (Invitrogen, MA5-17040, RRID:AB_2538512, mouse), TGFβ-1 (Abcam, ab9758, RRID:AB_296604, rabbit) and loading control α-tubulin (Sigma, T5168; RRID:AB_477579, mouse) or β-Actin (Sigma, A4700, clone AC-40, RRID:AB_476730, mouse). After three washes with 1× TBST (20 mM Tris, 150 mM NaCl, 0.1% Tween 20 detergent), membranes were incubated for 1 h at room temperature with horseradish peroxidase (HRP)-conjugated secondary antibodies (anti-mouse and anti-rabbit HRP-linked IgG antibodies; CST, #7076; RRID:AB_30924 and #7074; RRID:AB_2099233) diluted in 1× Roti Block buffer. Detection of proteins was performed using Amersham ECL Prime Western Blotting Detection Reagents (#RPN2236) and visualized on X-ray films (medical X-ray screen film blue sensitive, AGFA, CP-BU M).

### Flow cytometric analysis

Peritoneal exudate cells (PECs) were collected by injection of 10 mL of DPBS into the peritoneal cavity of mice, followed by withdrawal of the exudate. Spleen and tumor tissues were excised, and single-cell suspensions in DBPS were obtained by gently mincing the organs through 70-μm cell strainers. Erythrocytes were lysed using 1× RBC lysing buffer (Santa Cruz Biotechnology, sc-296258), then cells were incubated with mouse serum (Invitrogen, 10410) to block non-specific binding. After centrifugation at 300 × *g*, cells were resuspended in fluorescence-activated cell sorting (FACS) buffer (containing 1% Hi-FBS and 25 mM EDTA in DPBS) for subsequent surface marker staining. For macrophage polarization analysis, BMDMs were blocked with mouse serum, and then resuspended in FACS buffer. Cells were stained with panels of fluorophore-labeled antibodies from Thermo Scientific: anti-CD11b APC (1:320, 17-0114-82; RRID:AB_469346), anti-CD45R Alexa Fluor 488 (1:200, 53-0452-82; RRID:AB_469907), anti-F4/80 PE-Cy7 (1:200, 25-4801-82; RRID:AB_469653), anti-Ly6C Alexa Fluor 488 (1:800, 53-5932-82; RRID:AB_2574427), anti-Ly6G PE-Cy7 (1:400, 25-9668-82; RRID:AB_2811793), anti-CD11c APC (1:200, 17-0114-82, RRID:AB_469346), anti-MHCII PE (1:1000, 12-5321-82; RRID:AB_465928), anti-CD4 APC (1:320, 17-0042-82; RRID:AB_469323), anti-CD3 PE-Cy7 (1:100, 25-0032-82; RRID:AB_2815096), anti-CD8a Alexa Fluor 488 (1:200, 53-0081-82; RRID:AB_469897), anti-NK1.1 PE (1:160, 12-5941-82; RRID:AB_466050). For detection of CD206, cells were first stained with an unlabeled anti-CD206 antibody (1:100, AF2535, Bio-Techne; RRID:AB_2063012), followed by staining with Alexa Fluor 488 donkey anti-goat secondary antibody (1:1000, A32814, Invitrogen; RRID:AB_2762838). After doublet discrimination, a minimum of 1 × 10^5^ cells were analyzed using a BD Accuri C6 Plus System (Becton and Dickinson, Franklin Lakes, NJ, USA), equipped with 488- and 640-nm lasers. Data were processed using Accuri C6 Plus software. Gating strategies are provided in the Supplementary Materials (Supplementary Figs. S3, S5, S8).

### Paraffin section preparation and hematoxylin and eosin (H&E) staining

Liver and spleen specimens from NOD/SCID mice were fixed in 4% paraformaldehyde (PFA) at room temperature. Samples were dehydrated through gradient ethanol baths, cleaned in xylene, and embedded in paraffin at 60°C. Paraffin sections of 5-μm thickness were prepared using a microtome (Microm HM 360 Automated Microtome), floated in a 37°C water bath containing deionized water, and then floated onto clean glass slides, and left to warm at 37 °C overnight. These slides were used for hematoxylin and eosin (H&E) staining following a standard protocol. Briefly, the paraffin sections were deparaffinized in xylene (Sigma-Aldrich, 1330-20-7) for 20 min, followed by gradual hydration in 100% ethanol for 10 min, 95% ethanol for 10 min, 80% ethanol for 5 min, 70% ethanol for 3 min, 50% ethanol for 3 min, and finally rinsed in DPBS for 2 min. Subsequently, the slides were stained with hematoxylin for 1 min, differentiated in 1% hydrochloric ethanol for 5 s, blued in 1% ammonia for 10 s, and stained with eosin for 15 s. This was followed by dehydration in 70% ethanol for 1 min, 95% ethanol for 2 min, 100% ethanol for 2 min, and clearing in xylene for 5 min. Sections were then mounted with DPX medium (Sigma, BCBV8243) and covered with a cover glass to seal. Digital images were performed using a Nikon 90i Eclipse microscope at 20× magnification using the NIS-EAR software.

### Immunofluorescence (IF) staining and confocal imaging

Pieces of tumors were cryo-preserved in Tissue-Tek^®^ O.C.T. Compound (Sakura Finetek USA, Inc.). Then, 6-μm-thick sections were cut using a Cryostat CM-3050S (Leica Microsystems, Wetzlar, Germany) and mounted on SUPERFROST^®^ Plus Microscope Adhesion Slides (ThermoFisher Scientific, 10149870). The sections were stored at −80°C until use. Immunofluorescence (IF) staining of tumor sections was performed as described previously [44] with minor modifications. Briefly, the frozen sections were fixed with either 4% paraformaldehyde (PFA; for CD45 and Ly6G, CD45 and Ki67, CD45 and cleaved caspase-3) or cold acetone (Sigma-Aldrich Merck, 17124; for F4/80 and CD206) for 15 min. After fixation, the samples were washed twice in DPBS and permeabilized with 0.1% Triton X-100, 2% Hi-FBS and 1% bovine serum albumin (BSA) in DPBS for 30 min in a humidified chamber at room temperature (RT). The samples were then blocked with 2% Hi-FBS, 1% BSA in DPBS for 30 min at RT in a humidified chamber. Subsequently, samples were incubated with the corresponding primary antibodies at RT for 1 h using anti-CD45 (1:100; Abcam, ab10558; RRID:AB_442810, Rabbit), CD45 (1:100; Invitrogen, 14-0451-82; RRID:AB_467251, Rat) and anti-F4/80 (1:100; Bio-Rad, MCA497G; RRID:AB_872005, Rat), or overnight at 4 °C using anti-Ly6G (1:250; Bio-Legend, 127602; RRID:AB_1089180, Rat), anti-CD206 (1:500; Bio-Techne, AF2535; RRID:AB_2063012, Goat), anti-Ki67 (1:250; Abcam, ab16667; RRID:AB_302459, Rabbit) and anti-cleaved caspase-3 (1:200; CST, #9661; RRID:AB_2341188, Rabbit). After twice washing with DPBS, slides were incubated with the appropriate Alexa Fluor-conjugated secondary antibody at RT for 1 h (1:1000 diluted in blocking buffer), including Alexa Fluor 555 Goat anti-Rat (Invitrogen, A21434; RRID:AB_2535855) for F4/80, Alexa Fluor 488 Donkey anti-Goat (Invitrogen, A32814; RRID:AB_2762838) for CD206, Alexa Fluor 568 Goat anti-Rabbit (Invitrogen, A11011; RRID:AB_10078418) for CD45 (Rabbit), Alexa Fluor 555 Goat anti-Rat (Invitrogen, A21434; RRID:AB_2535855) for CD45 (Rat), Alexa Fluor 488 Donkey anti-Rat (Invitrogen, A21208; RRID:AB_2535794) for Ly6G, and Alexa Fluor 488 Donkey anti-Rabbit (Invitrogen, A21206; RRID:AB_2535792) for Ki67 and cleaved caspase-3. Slides were mounted with VECTASHIELD^®^ PLUS Antifade Mounting Medium with DAPI (Vector Laboratories, H-2000, Newark, CA, USA) and covered with cover glasses. Images of 10~20 non-overlapping optical fields covering the surface of the sections were acquired using a confocal laser scanning microscope (Leica TCS SP8, Leica Microsystems) equipped with a ×40 oil objective. The number of positive cells and cell fluorescence intensity of individual positive cells were analyzed using ImageJ/FIJI Software.

### In-situ transcriptomic analysis

In situ detection of *Il10*, *Ifng*, *Mx1*, and *Tnf* mRNA expression was performed using the RNAscope® Multiplex Fluorescent Reagent Kit v2 (Bio-Techne, 323100, Oxford, UK) according to the manufacturer’s instructions, with minor modifications [45]. A positive control probe targeting the housekeeping genes Polymerase II Subunit A (*Polr2a*), Peptidylpropyl isomerase B (*PPIB*) and Ubiquitin C (*UBC*) (Bio-Techne, 320881) and a negative control probe targeting the *DapB* mRNA from the *Bacillus subtilis* strain SMY (Bio-techne, 320871) were used to assess mRNA quality in the samples. Briefly, 6-μm O.C.T. tumor sections were fixed with pre-chilled 4% PFA at 4°C for 15 min and dehydrated in sequential ethanol immersions (50%, 70%, and 100%) for 5 min at RT. Sections were then treated with RNAScope Hydrogen Peroxide for 10 min and RNAScope Protease IV (Bio-techne) at RT for 30 min. Subsequently, samples were incubated with a corresponding probe mixture (1 volume of Channel 2 probe and 50 volumes of Channel 1 probe) at 40°C for 2 h. The sections were washed with 1× wash buffer and incubated in 5× SSC buffer at RT overnight, and then again washed with 1× wash buffer. Samples were hybridized with AMP solutions by incubation with Multiplex FL v2 Amp 1, Multiplex FL v2 Amp 2, and Multiplex FL v2 Amp 3 at 40°C for 30 min. Subsequently, the samples were developed with HRP-C1 and HRP-C2 signals by incubation with Multiplex FL v2 HRP-C1 and Multiplex FL v2 HRP-C2, respectively, at 40°C for 15 min. The signal of target and control probes was detected after incubation with the fluorescent dyes Opal 520 and Opal 570 (1:750 for target probes; 1:1500 for control probes, NEL810001KT, PerkinElmer, Waltham, MA, USA) at 40°C for 30 min followed by incubation with Multiplex FL v2 HRP blocker at 40°C for 15 min. All incubation steps were performed at 40°C in the HybEZ™ II Oven (Bio-techne, 321719). Nuclei were counterstained using VECTASHIELD^®^ PLUS Antifade Mounting Medium with DAPI (Vector Laboratories, H-2000) and mounted with cover glasses. Images were captured using a confocal laser scanning microscope (Leica TCS SP8, Leica Microsystems) equipped with a ×63 oil objective. The number of positive cells in each five (for *Il10* and *Ifng*) and ten (for *Mx1* and *Tnf*) fields of view were counted with each colorful spot representing a single mRNA. Scoring was based on a scale from 1 to 2, where 1 represented cells with 1~3 spots, whereas 2 represented those with 4~9 spots. Expression levels of each target probe were quantitatively determined using an H-score, calculated as: H-score = (percentage of positive cells with 1~3 spots × 1) + (percentage of cells with positive 4~9 spots × 2).

### RNAscope™ combined with IF

For detecting the co-localization of the target mRNA probe (*Ifng*) with leucocyte marker CD45 or macrophage marker F4/80, the RNA-Protein Co-Detection Ancillary Kit (323180, Bio-Techne) according to manufacturers’ instructions with minor modifications was used as described previously [45]. Briefly, after the fixation and dehydration as outlined in the RNAscope™ assay, the tumor sections were incubated with the corresponding primary F4/80 antibody at 4°C overnight. Subsequently, the sections were washed in 0.1% Triton X-100 in PBS and fixed in 4% PFA for 30 min at RT. The sections were then pre-treated with RNAScope Hydrogen Peroxide for 10 min and RNAScope Protease IV at RT for 30 min, followed by incubation with the corresponding probe mixture at 40°C for 2 h. The subsequent hybridization steps and probe signal detection with fluorescent dyes were performed as described in the RNAscope™ assay. This was followed by a 30-min incubation with the secondary antibody Alexa Fluor 647 Goat anti-Rat (1:1000, Invitrogen, A21247) at RT in a humidified chamber to detect CD45 and F4/80. Sections were counterstained with VECTASHIELD^®^ PLUS Antifade Mounting Medium with DAPI and covered with cover glasses. Images were acquired using a confocal laser scanning microscope (Leica TCS SP8, Leica Microsystems) equipped with a ×63 oil objective.

### MakA production

Cloning, overexpression, and purification of MakA were performed as described previously [10]. Endotoxin contamination was assessed using the Pierce™ Rapid Gel Clot Endotoxin Assay Kit (Thermo Scientific™; A43879). The endotoxin level in the final protein purification was determined to be <0.03 EU/ml.

### Statistical analysis

Data are presented as mean ± standard error of the mean (SEM) and were visualized using GraphPad Prism version 10.4.0 (GraphPad Software, Inc). In vitro data presented as single data points are collected from at least three independent experiments. In vivo experiments were performed twice, if not indicated differently. Each specific tissue analysis was performed on organs of 3–5 mice. Statistical analyses were performed using GraphPad Prism version 10.4.0. Differences among more than two experimental groups were evaluated using a one-way ANOVA followed by Dunnett’s multiple comparisons test, whereas comparisons between two groups were analyzed using unpaired two-tailed *t*-test. Differences among two experimental groups over time were evaluated using two-way ANOVA. Significant differences were defined as *P* values less than 0.05. *P* values not significant are indicted as *P* > 0.05, *P* values smaller than 0.001 are indicated as *P* < 0.001. For all other *P* values, the exact values are indicated.

### Ethics statement

Animal experiments were carried out according to the guidelines set by the Umeå Regional Animal Ethic Committee (Umeå Regionala Djurförsöksetiska Nämnd), Approval No. A42-2019.

## Supplementary information

Supplementary Document 1 - Suppl Figures (Without highlights)

Supplementary Document 2 - Original Western Blots (Without highlights)

## Data Availability

All data are available in the main text or the supplementary materials. Raw data for all experiments either can be found in Supplementary Information or are available from the corresponding author(s) upon reasonable request. Requests for further information and resources should be directed to and will be fulfilled by the lead contact Saskia F. Erttmann (saskia.erttmann@umu.se) or Sun Nyunt Wai (sun.nyunt.wai@umu.se). This paper does not report original code. This study did not generate new unique reagents.
