# Supplementary material for: A bacterial toxin as a novel anti-cancer drug modulating the tumor-microenvironment
Source: Cell Death Dis. 2025 Dec 1;16(1):874. doi: 10.1038/s41419-025-08219-2 (PMC12669713; doi:10.1038/s41419-025-08219-2)
Supplement: Supplementary file 1 — Supplementary Document 1 - Suppl Figures (Without highlights) [file 41419_2025_8219_MOESM1_ESM.pdf]

A bacterial toxin as a novel anti-cancer drug modulating the tumor-microenvironment

Running title: A bacterial toxin as novel anti-cancer therapeutic

Lingyu Li[1], Pauline Evain[1], Michael Timothy Phillips[1], Maria Lopez Chiloeches[1], Anna Bergonzini[1], Teresa Frisan[1], Sun Nyunt Wai[1,2,*], Saskia Friederike Erttmann[1,3,*]

[1]Department of Molecular Biology, Umeå Centre for Microbial Research (UCMR), Umeå University, SE-90187, Umeå, Sweden
[2]The Laboratory for Molecular Infection Medicine Sweden (MIMS), Umeå University, SE-90187, Umeå, Sweden
[3]Laboratory of Infection Oncology, Institute of Clinical Molecular Biology, University of Kiel and University Hospital Schleswig Holstein (UKSH), 24105, Kiel, Germany

*These authors contribute equally

Correspondence: saskia.erttmann@umu.se and sun.nyunt.wai@umu.se

Supplementary Information

Supplementary Document S1. Supplementary Figures.

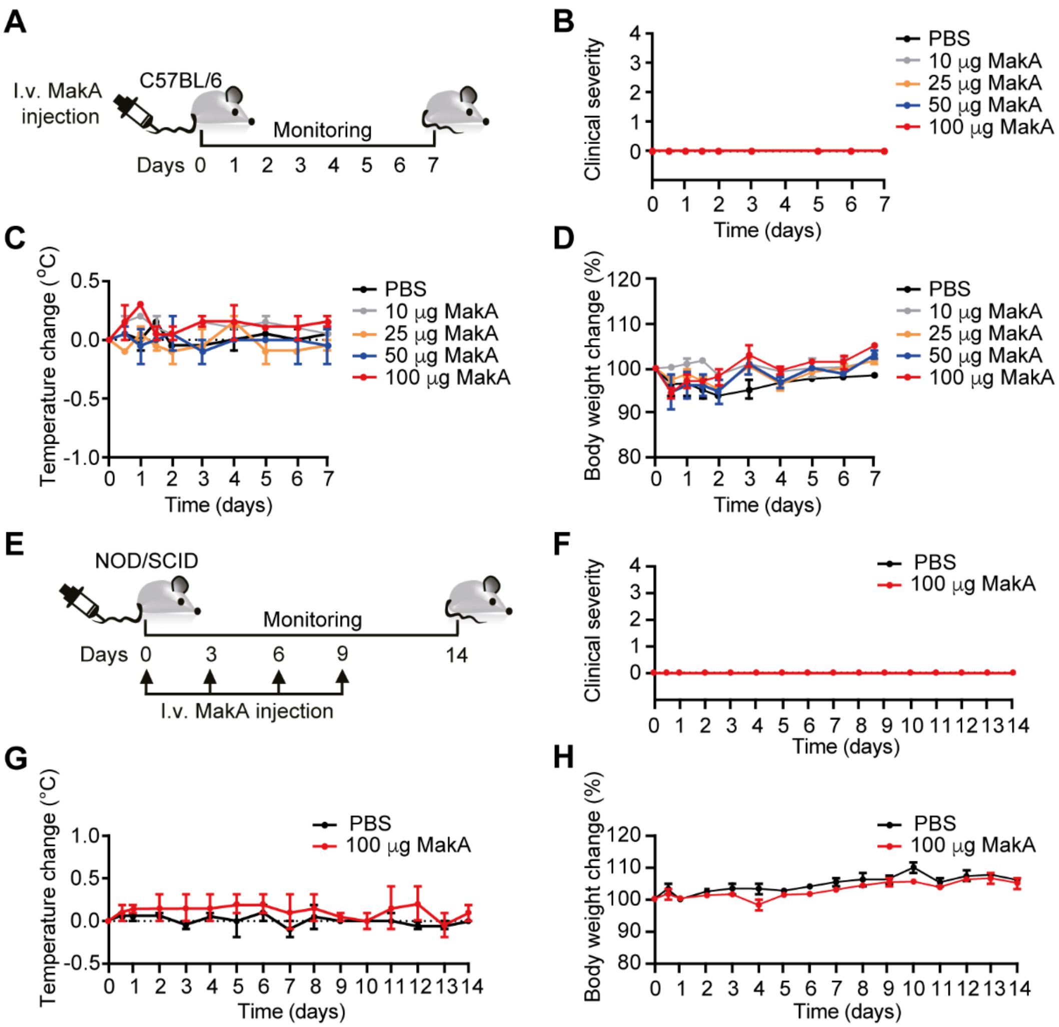

Supplementary Fig. S1. Determination of non-toxicity of MakA during systemic administration. Related to Fig. 1.

(**A**) Experimental layout of monitoring C57BL/6 mice intravenously (*i.v.*) injected with 10, 25, 50, or 100 µg MakA (or PBS) over a period of 7 days, used in B–D. (**B**) Clinical severity. (**C**) Subcutaneous body temperature change in percent. (**D**) Body weight change in percent. Data in B–D are presented as means ± standard error of the mean (SEM) from one experiment with n=3 mice per group. (**E**) Experimental layout of monitoring NOD/SCID mice *i.v.* injected with 100 µg MakA (or PBS) on day 0, 3, 6 and 9, over a total period of 14 days, used in F–H. (**F**) Clinical severity. (**G**) Subcutaneous body temperature change in percent. (**H**) Body weight change in percent. Data in F–H are presented as means ± SEM from one experiment with n=3 mice per group.

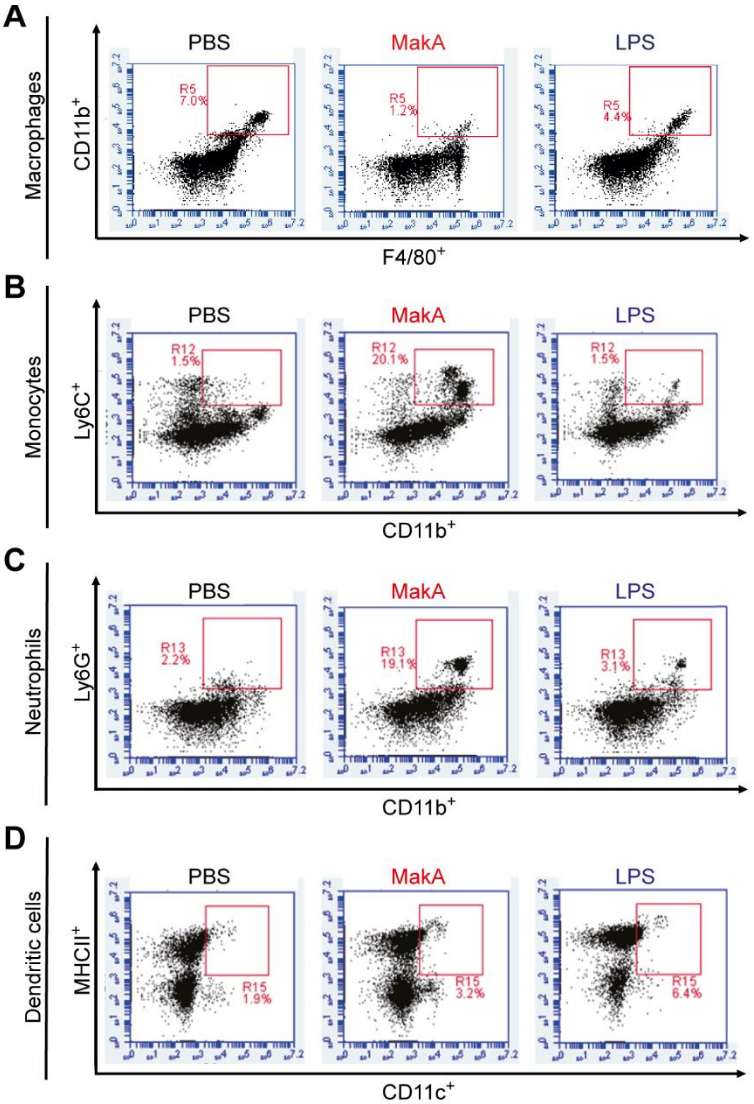

Supplementary Fig. S2. Effect of MakA on immune cell populations in the peritoneal cavity. Related to Fig. 1.

(**A-D**) C57BL/6 mice were intraperitoneally injected with 2.5 mg kg^{-1} MakA, 10 mg kg^{-1} lipopolysaccharide (LPS) or PBS. After 6 h, different cell populations in the peritoneal cavity were analyses by flow cytometry. Representative flow cytometric analyses of (**A**) macrophages ($CD11b^+$ $F4/80^+$ cells), (**B**) monocytes ($CD11b^+$ $Ly6C^+$ cells), (**C**) neutrophils ($CD11b^+$ $Ly6C^+$ cells), and (**D**) dendritic cells ($CD11c^+$ $MHCII^+$ cells), respectively, in total peritoneal exudate cells.

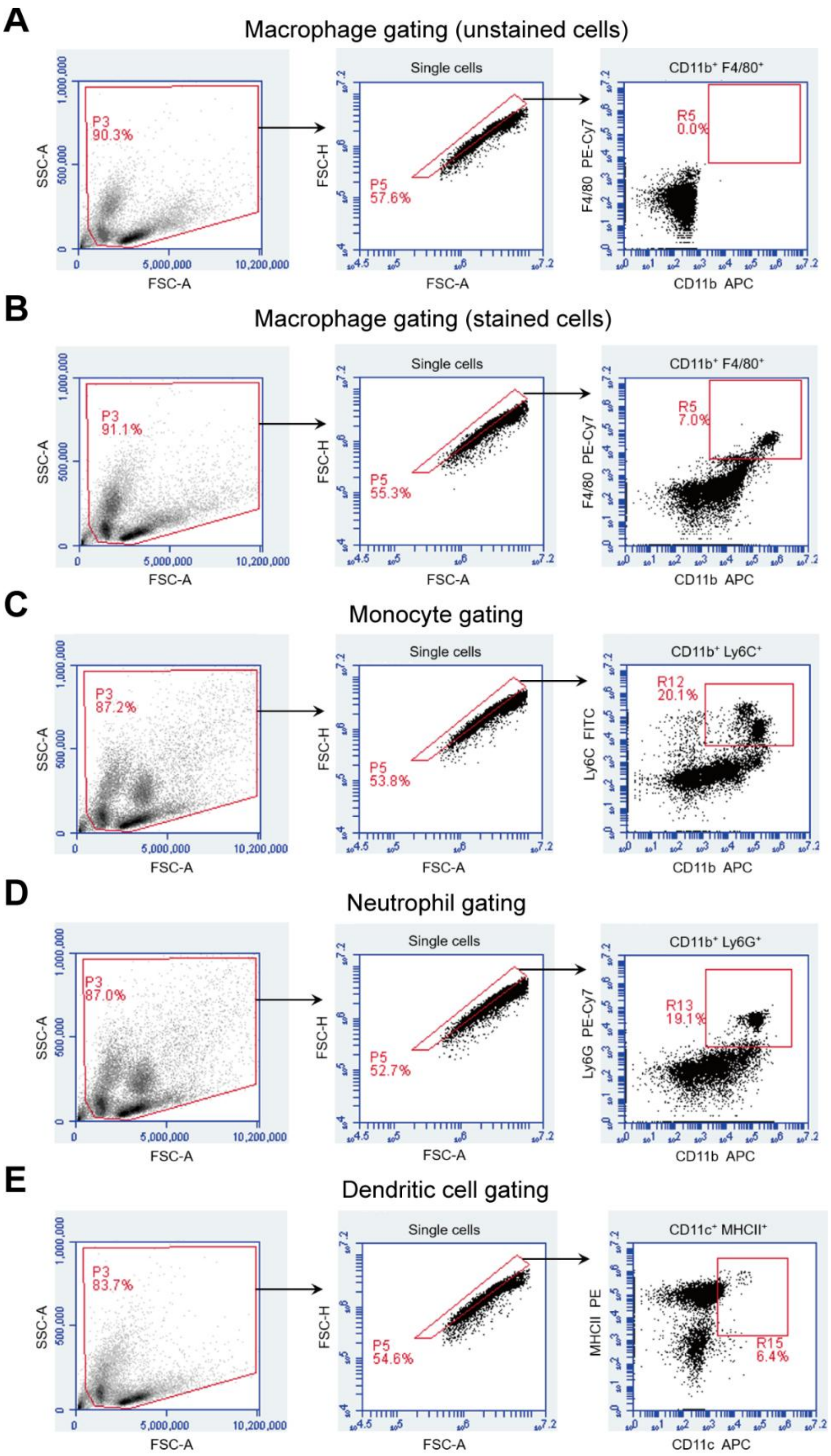

Supplementary Fig. S3. Gating strategies of flow cytometric analyses. Related to Fig. 1. (**A-D**) Cells were gated using FSC-A vs SSC-A. Doublets were excluded using FSC-A vs FSC-H. (**A**) Unstained and (**B**) stained cells were gated by $CD11b^+$ $F4/80^+$ (APC/PE-Cy7) for macrophages. (**C**) Cells were gated by $CD11b^+$ $Ly6C^+$ (APC/FITC) for monocytes, (**D**) $CD11b^+$ $Ly6G^+$ (APC/PE-Cy7) for neutrophils, and (**E**) $CD11c^+$ $MHCII^+$ (APC/PE) for dendritic cells.

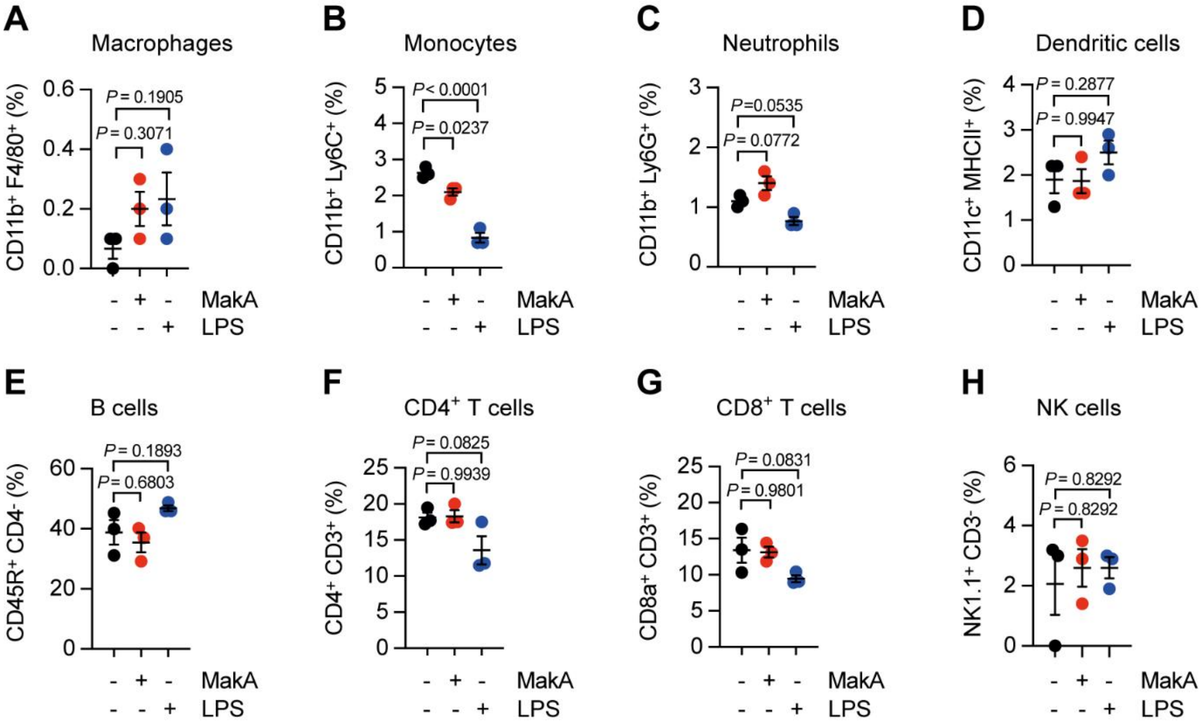

Supplementary Fig. S4. MakA lacks the ability to mediate immune cell infiltration in the spleen. Related to Fig. 1.

(**A-H**) C57BL/6 mice were intraperitoneally injected with 2.5 mg kg^{-1} MakA, 10 mg kg^{-1} lipopolysaccharide (LPS), or PBS. Flow cytometric analysis was performed to determine immune cell infiltration into the spleens 6 hours post-injection. (**A**) macorphages ($CD11b^{+}$ $F4/80^{+}$), (**B**) monocytes ($CD11b^{+}$ $Ly6C^{+}$), (**C**) neutrophils ($CD11b^{+}$ $Ly6G^{+}$), (**D**) dendritic cells ($CD11c^{+}$ $MHCII^{+}$), (**E**) B cells ($CD45R^{+}$ $CD4^{-}$), (**F**) $CD4^{+}$ T cells ($CD4^{+}$ $CD3^{+}$), (**G**) $CD8^{+}$ T cells ($CD8a^{+}$ $CD3^{+}$) and (**H**) NK cells ($NK1.1^{+}$ $CD3^{-}$). Data are presented as means ± SEM, n=3 mice per group from one experiment.

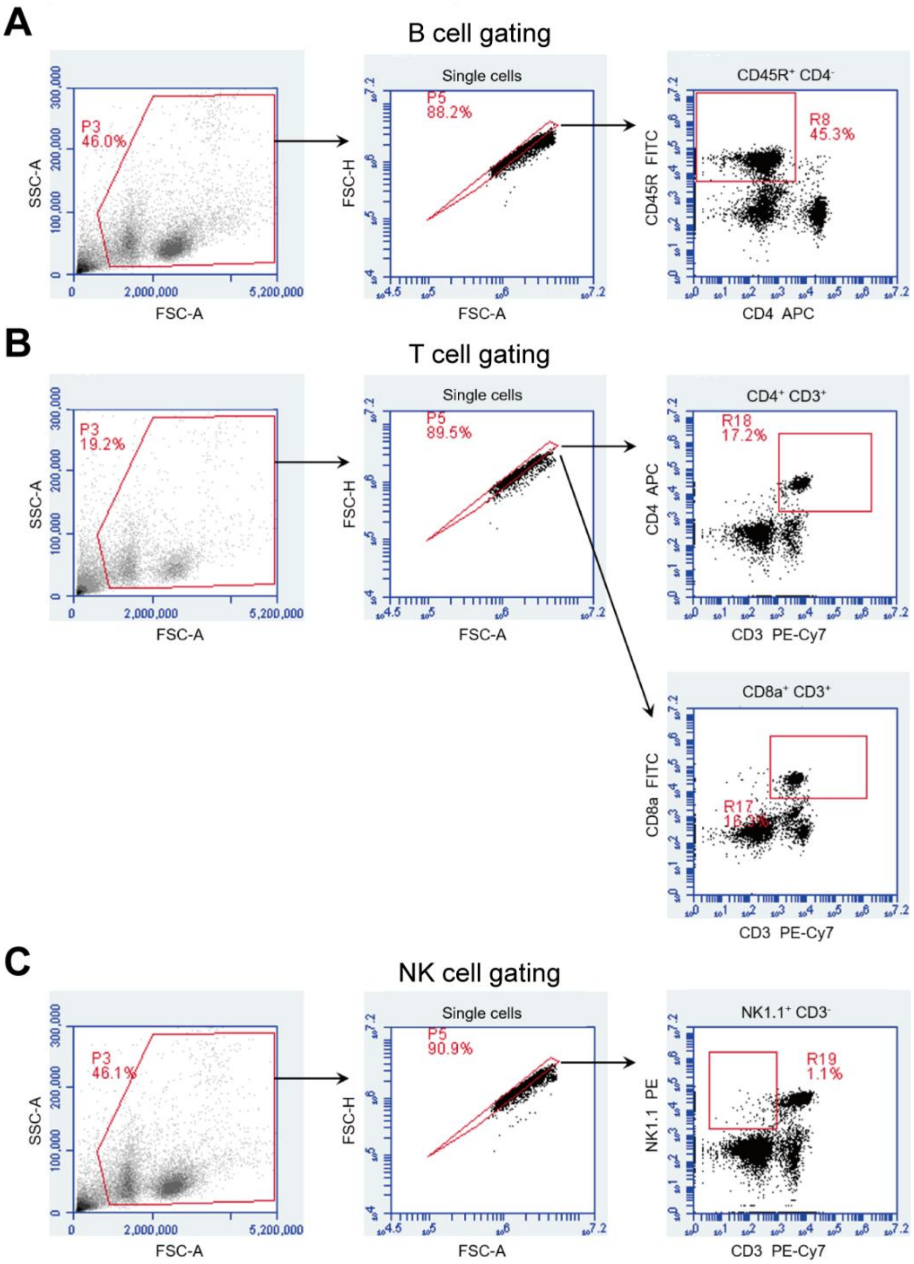

Supplementary Fig. S5. Gating strategies of flow cytometric analyses. Related to Fig. 1. (**A**-**C**) Cells were gated using FSC-A vs SSC-A. Doublets were excluded using FSC-A vs FSC-H. (**A**) Stained cells were gated by CD45R$^+$ CD4$^-$ (FITC/PE-APC) for B cells, (**B**) CD4$^+$ CD3$^+$ or CD8a$^+$ CD3$^+$ (APC/PE-Cy7) for CD4$^+$ and CD8$^+$ T cells, respectively, and (**C**) NK1.1$^+$ CD3$^-$ (PE/PE-Cy7) for NK cells.

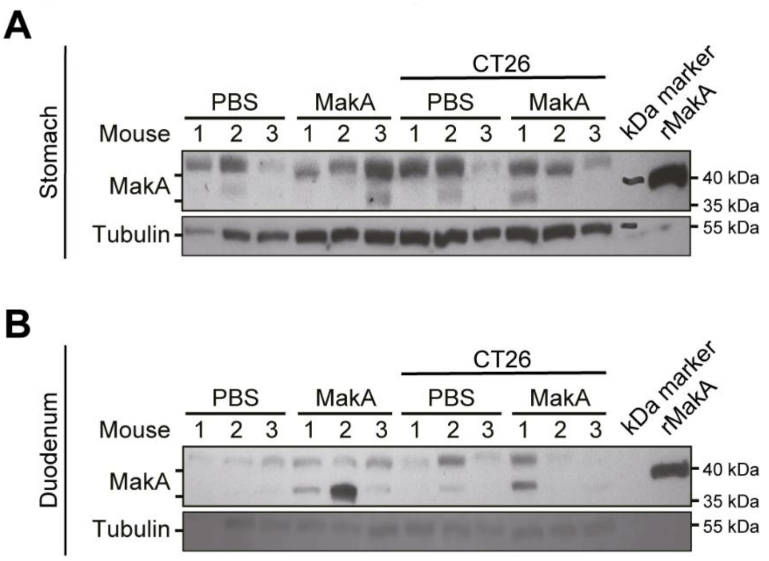

Supplementary Fig. S6. MakA does not accumulate in the stomach or duodenum of mice. Related to Fig. 2 and 3.

(**A**, **B**) Immunoblotting for MakA in tissue lysates of A the stomach and B duodenum of NOD/SCID mice injected with CT26 cells (or not) and then treated three times with MakA or PBS. Representative results from tissue lysates of three mice per group are depicted. α-Tubulin served as loading control. rMakA denotes recombinant MakA control; kDa marker indicates the protein ladder lane.

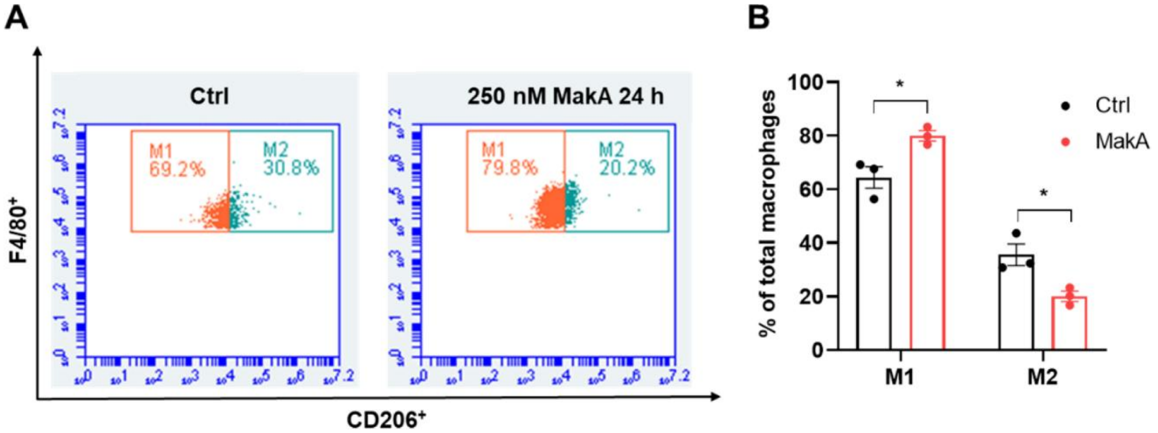

Supplementary Fig. S7. MakA induces the polarization of M1-like macrophages. Related to Fig. 4.

(A) Representative flow cytometry analyses of M1-like (F4/80^{+} CD206^{-}) and M2-like (F4/80^{+} CD206^{+}) macrophages in murine bone marrow-derived macrophages (BMDMs) treated with or without MakA. (**B**) Flow cytometric quantification in percent of M1- and M2- like macrophages in BMDMs treated with or without MakA. Results are depicted as means ± SEM of three mice per group.

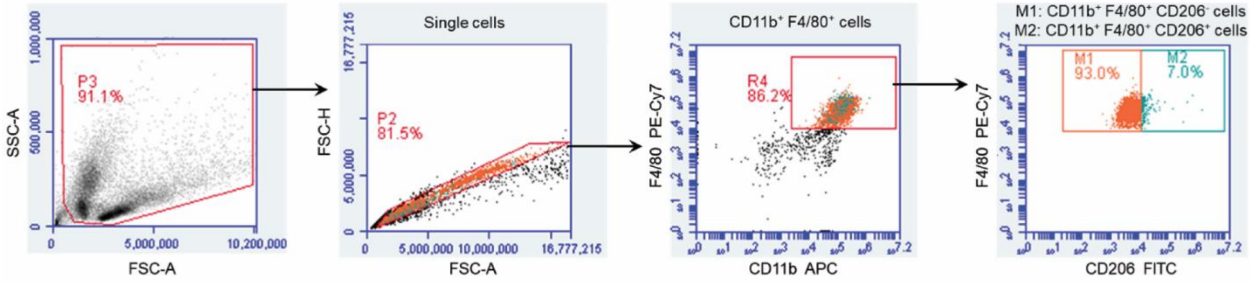

Supplementary Fig. S8. Gating strategies of flow cytometric analyses. Related to Fig. 4 and Supplementary Fig. S7.

Cells were gated using FSC-A vs SSC-A. Doublets were excluded using FSC-A vs FSC-H. Stained cells were gated by F4/80$^+$ CD11b$^+$ (PE-Cy7/APC) for total macrophages, and F4/80$^+$ CD206$^-$, F4/80$^+$ CD206$^+$ (PE-Cy7/FITC) for M1- and M2- like macrophages, respectively.

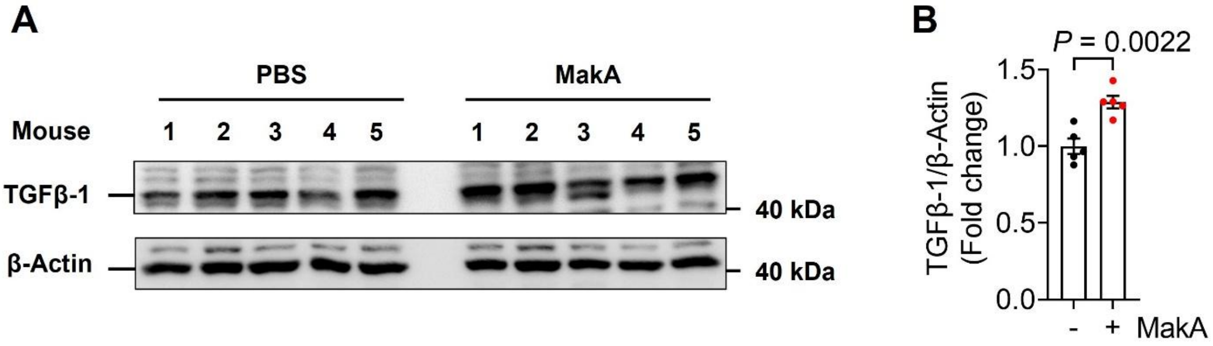

Supplementary Fig. S9. MakA affects the protein level of cytokines in tumor tissues. Related to Fig. 5.

(**A**) Immunoblotting for TGFβ-1 in tumor tissue lysates of NOD/SCID mice injected with CT26 cells and then treated three times with MakA or PBS. (**B**) Quantification of the protein level of TGFβ-1 in tumor tissue. β-Actin served as loading control. Representative results from tissue lysates of five mice per group are depicted. Results are depicted as means ± SEM.

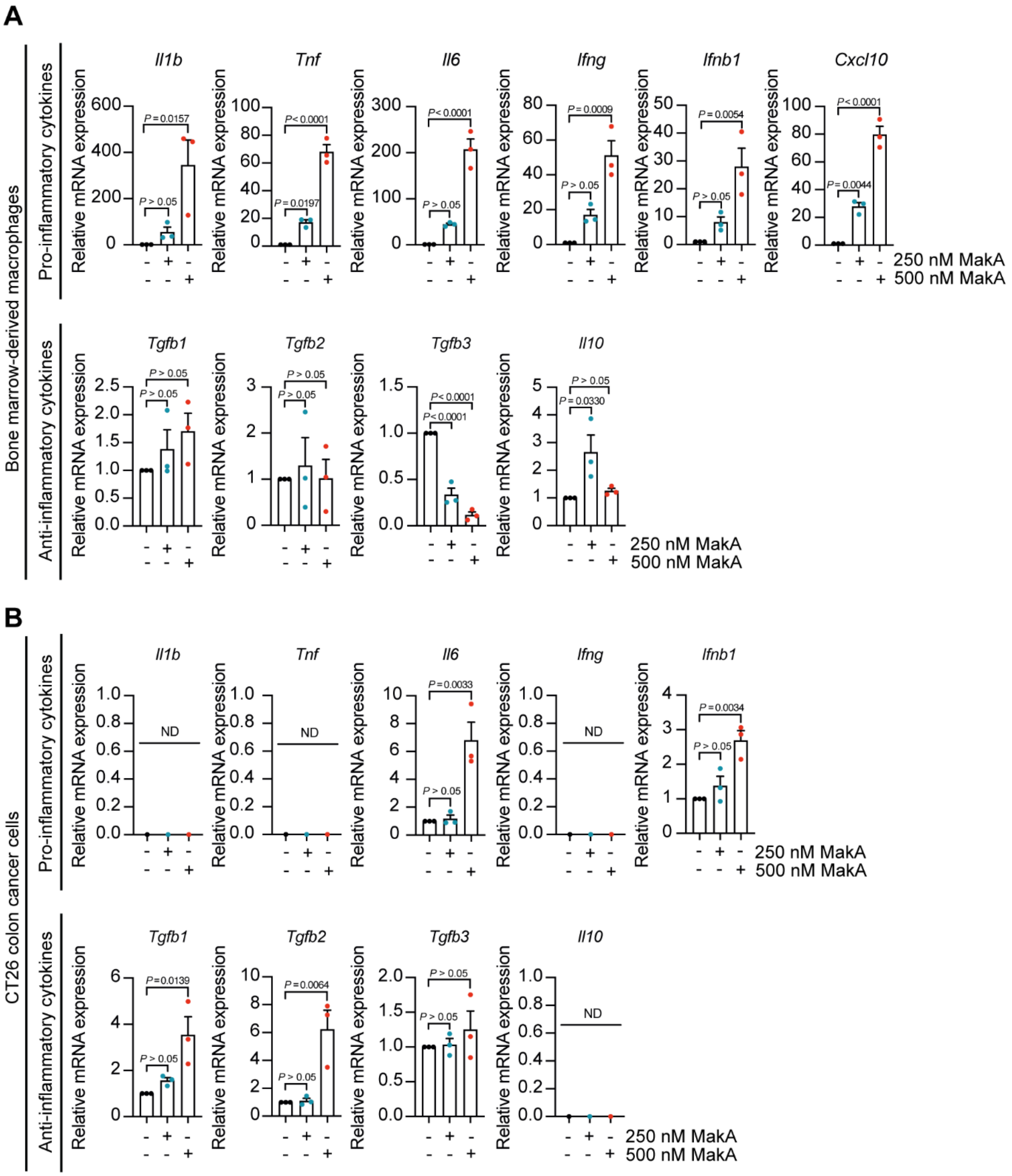

Supplementary Fig. S10. MakA drives cytokine gene expression in primary macrophages but not in cancer cells. Related to Fig. 5.

(**A**) Pro- and anti-inflammatory cytokine gene expression in murine bone marrow-derived macrophages (BMDMs) after 12-h treatment with 250 and 500 nM MakA. (**B**) Pro- and anti-inflammatory cytokine gene expression in CT26 cancer cells after 12-h treatment with 250 and 500 nM MakA. Results are depicted as means ± SEM of three independent experiments. ND (not detectable) indicates RT-qPCR results of mRNA levels below the detection limit.
