# Supplementary material for: A bacterial toxin as a novel anti-cancer drug modulating the tumor-microenvironment
Source: Cell Death Dis. 2025 Dec 1;16(1):874. doi: 10.1038/s41419-025-08219-2 (PMC12669713; doi:10.1038/s41419-025-08219-2)
Supplement: Supplementary file 2 — Supplementary Document 2 - Original Western Blots (Without highlights) [file 41419_2025_8219_MOESM2_ESM.pdf]

A bacterial toxin as a novel anti-cancer drug modulating the tumor-microenvironment

Running title: A bacterial toxin as novel anti-cancer therapeutic

Lingyu Li[1], Pauline Evain[1], Michael Timothy Phillips[1], Maria Lopez Chiloeches[1], Anna Bergonzini[1], Teresa Frisan[1], Sun Nyunt Wai[1,2,*], Saskia Friederike Erttmann[1,3,*]

[1]Department of Molecular Biology, Umeå Centre for Microbial Research (UCMR), Umeå University, SE-90187, Umeå, Sweden
[2]The Laboratory for Molecular Infection Medicine Sweden (MIMS), Umeå University, SE-90187, Umeå, Sweden
[3]Laboratory of Infection Oncology, Institute of Clinical Molecular Biology, University of Kiel and University Hospital Schleswig Holstein (UKSH), 24105, Kiel, Germany

*These authors contribute equally
Correspondence: saskia.erttmann@umu.se and sun.nyunt.wai@umu.se

Supplementary Information

Supplementary Document S2. Original full length Western blots.

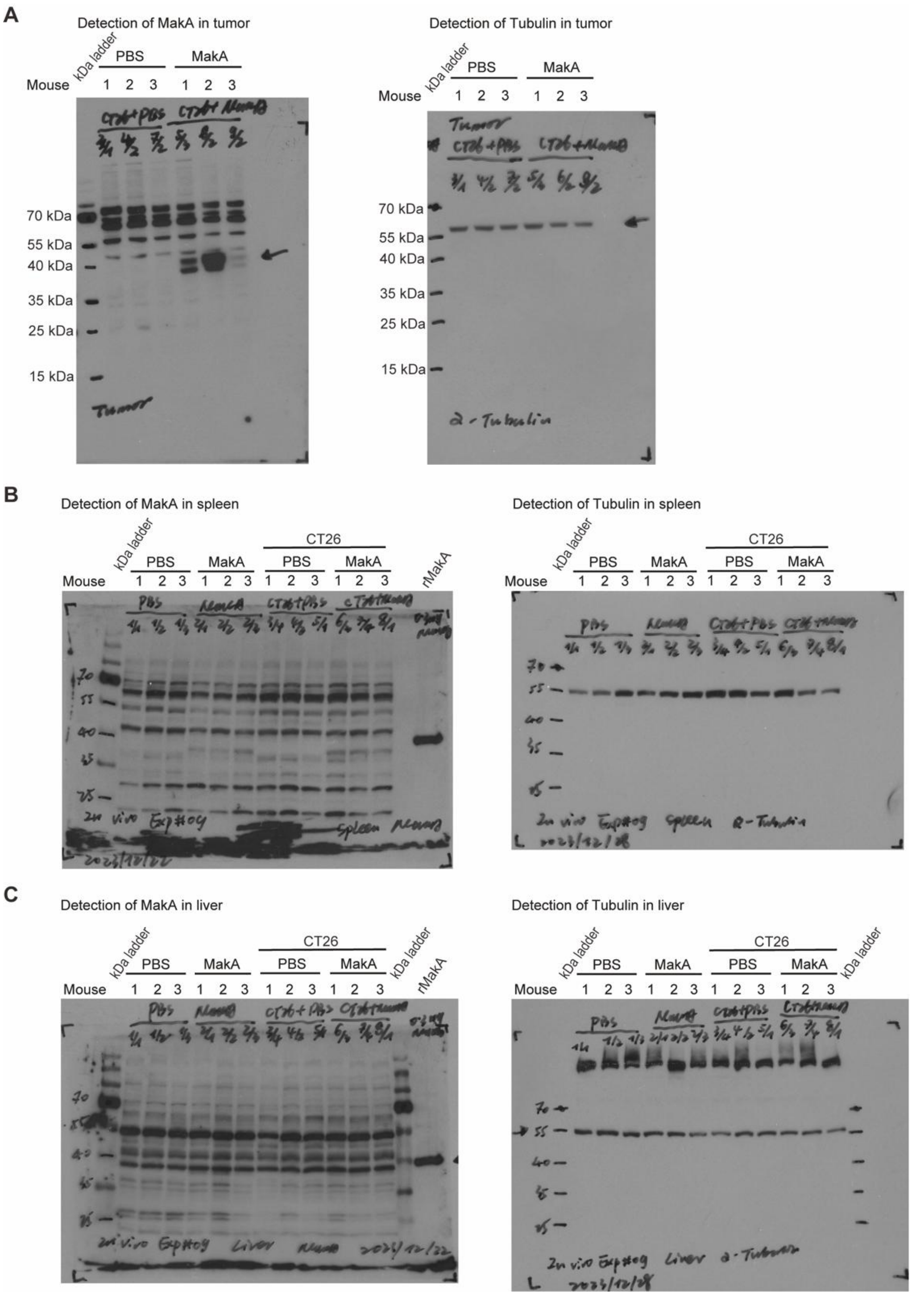

Supplementary Fig. S11. Original Western blots related to Fig. 2. (**A**) Original immunoblots of MakA and a-Tubulin in tumor tissue lysates presented in Fig. 2E. (**B**) Original immunoblots of MakA and a-Tubulin in spleen lysates presented in Fig. 2J. (**C**) Immunoblots of MakA and a-Tubulin in liver lysates presented in Fig. 2K. In A-C three mice per group, with or without MakA treatment in the presence or absence of CT26 cells, are shown. rMakA denotes recombinant MakA control; kDa marker indicates the protein ladder lane.

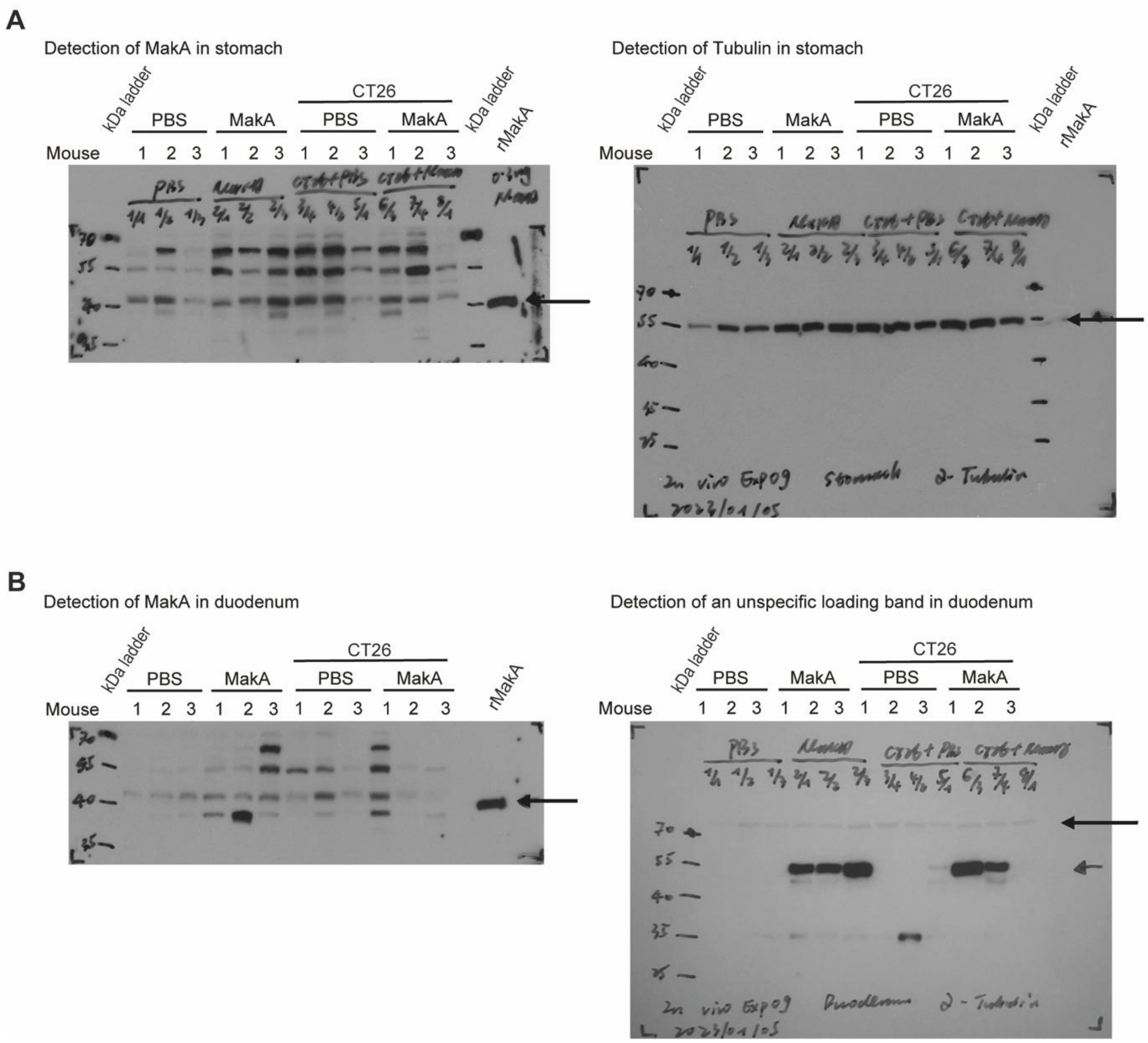

Supplementary Fig. S12. Original Western blots related to Supplementary Fig. S6. (**A**) Original immunoblots of MakA and α-Tubulin in stomach lysates presented in Supplementary Fig. S6A. (**B**) Original immunoblots of MakA and an unspecific loading band in duodenum lysates presented in Supplementary Fig. S6B. In A and B three mice per group, with or without MakA treatment in the presence or absence of CT26 cells, are shown. rMakA denotes recombinant MakA control; kDa Marker indicates the protein ladder lane.

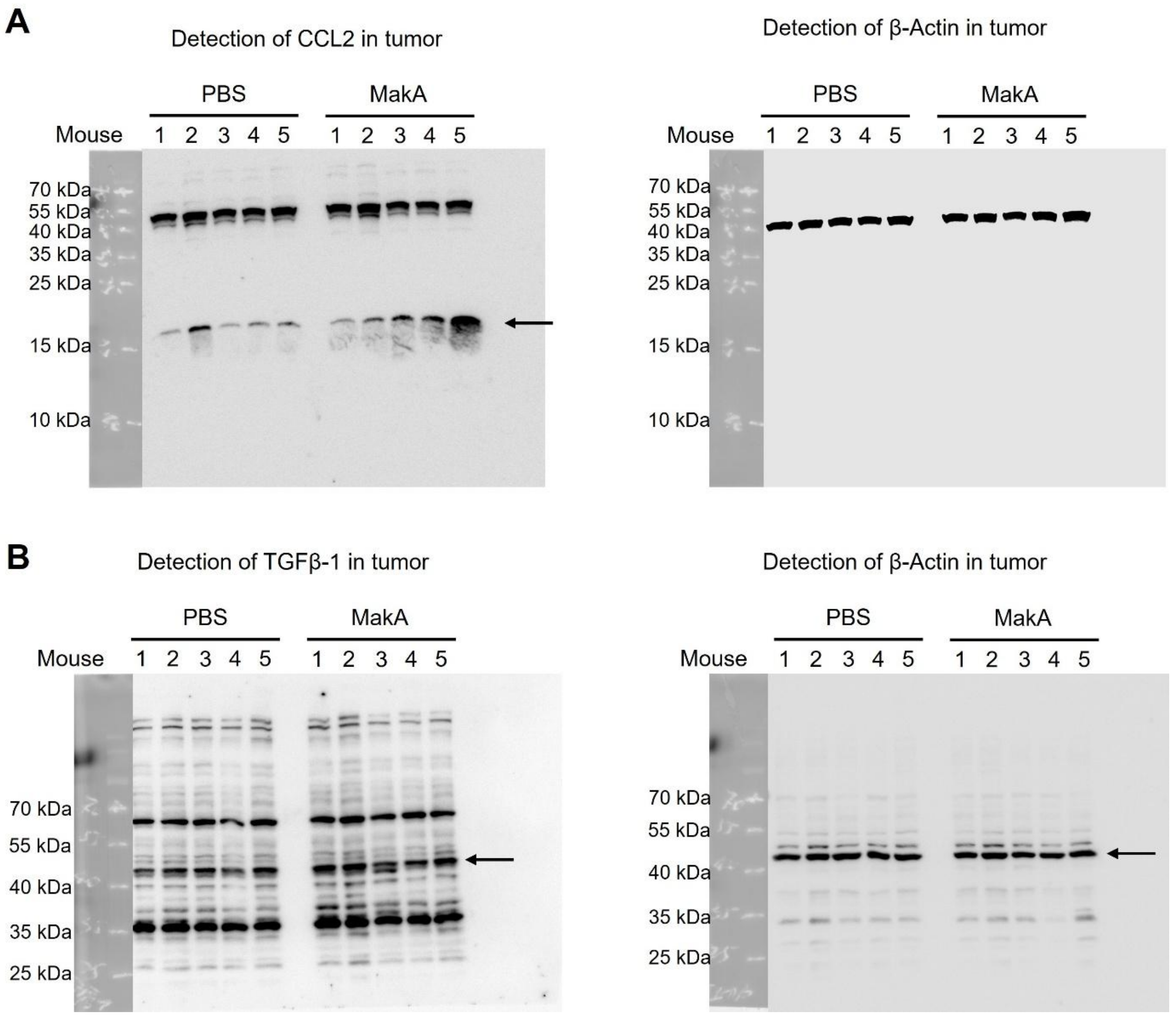

Supplementary Fig. S13. Original Western blots related to Fig. 5 and Supplementary Fig. S9. (**A**) Original immunoblots of CCL2 and β-Actin in tumor tissue lysates presented in Fig. 5B. (**B**) Original immunoblots of TGFβ-1 and β-Actin in tumor tissue lysates presented in Supplementary Fig. S9A. In A-B, five mice per group, with or without MakA treatment in the presence of CT26 cells, are shown.
